# Rare-Earth Elements at the Interface of Chemistry and Cancer Therapy

**DOI:** 10.3390/molecules31081264

**Published:** 2026-04-11

**Authors:** Christian Goldiș, Nicoleta Anamaria Pașcalău, Roxana Racoviceanu, Tamara Maksimovic, Mihaela Jorgovan, Elisabeta Atyim, Oana Bătrîna, Marius Mioc, Codruța Șoica

**Affiliations:** 1 Faculty of Medicine, “Victor Babeș” University of Medicine and Pharmacy, 2 Eftimie Murgu Square, 300041 Timisoara, Romania; christian.goldis@student.umft.ro; 2 Department of Psycho Neuroscience and Recovery, Faculty of Medicine and Pharmacy, University of Oradea, 410087 Oradea, Romania; 3 Faculty of Pharmacy, “Victor Babeș” University of Medicine and Pharmacy, 2 Eftimie Murgu Square, 300041 Timisoara, Romania; tamara.maksimovic@umft.ro (T.M.); mihaela.coban@umft.ro (M.J.); elisabeta.atyim@umft.ro (E.A.); oana.esanu@umft.ro (O.B.); marius.mioc@umft.ro (M.M.); codrutasoica@umft.ro (C.Ș.); 4 Research Centre for Experimental Pharmacology and Drug Design (X-Pharm Design), “Victor Babeș” University of Medicine and Pharmacy, 2 Eftimie Murgu Square, 300041 Timisoara, Romania

**Keywords:** lanthanide, yttrium, scandium, anticancer, metal-complexes, nanoparticles

## Abstract

Rare-earth elements (REEs), which include the entire lanthanide series together with scandium and yttrium, have unique electronic configurations and coordination chemical properties that provide them with special magnetic, optical, and redox abilities. Generally used for diagnostic imaging and theranostic applications, increasing evidence emphasizes their potential as direct anticancer agents. This review aims to present a thorough investigation of the studies published in the last ten years that focus on the intrinsic anticancer properties of REE-based molecular complexes and nanostructures, without discussing their recognized imaging functions. Rare-earth compounds exhibit selective cytotoxicity against malignant cells via mechanisms that mainly include modulations in the generation of reactive oxygen species, mitochondrial dysfunctions, interaction with DNA molecules, apoptosis, and ferroptosis induction, as well as radiosensitization. Molecular complexes that are based on the trivalent coordination chemistry of REEs enable them to target biomolecules like DNA and serum albumin. Nanostructured systems, on the other hand, render tumors more responsive to treatment by improving the cellular uptake, enabling surface functionalization, and controlling ROS generation. Terbium, thulium, yttrium, scandium, ytterbium, cerium, erbium, dysprosium, and europium show different levels of anticancer activity in both in vitro and in vivo cancer models. They often exert more toxicity in tumor cells than in normal tissues, thus exhibiting selective anticancer effects. The findings collectively underscore the therapeutic potential of REE-based compounds as novel metal-based anticancer agents and advocate for additional mechanistic and translational research to enhance their clinical applicability.

## 1. Introduction

Rare-earth elements (REEs) are a group of 17 chemical elements that include the 15 lanthanides (lanthanoids, according to IUPAC) as well as scandium (Sc) and yttrium (Y); although they are formally group 3 transition metals, they exhibit comparable ionic radii, trivalent chemistry, coordination characteristics, and frequent coexistence with lanthanides in mineral deposits [1]. In fact, some of the most significant properties shared by REEs include: (1) a greater reactivity compared to transition metals and more similar to group 2 elements, (2) a wide range of coordination numbers as well as coordination properties determined by the steric parameters of ligands rather than crystal-field effects, (3) the ability to form labile ionic complexes prone to facile ligand exchange, (4) the formation of hydroxides insoluble at neutral pH, (5) a preferred +3 oxidation state, and (6) a preference for anionic ligands containing highly electronegative atoms [2]. REEs exhibit a [Xe]6s^2^4f^n^ electronic configuration where 4f orbitals are gradually filled but strongly shielded by the completely filled 5s and 5p orbitals, a phenomenon known as lanthanide contraction; this limits the spatial expansion of the 4f orbitals and subsequently gradually decreases the ionic radii across REEs [3]. Collectively, the chemical configuration combined with high coordination numbers and preferential ionic bonding provides REEs with unique optical, magnetic, and redox properties that trigger particular technological and biomedical applications [4]. A comparison of their main electronic, structural, and physicochemical properties is provided in Table 1.

The term “rare” is rather improper because REEs are not actually scarce but can only be found as compounds instead of pure metals, and are difficult to separate and purify. Moreover, they were considered exotic elements when they were first discovered at the end of the 18th century. Additionally, the term “earth” is an archaic name for various minerals soluble in acids and resistant to heating, usually metal oxides, found in the Earth’s crust; however, the name “rare-earth elements” has become established in the specialized literature.

Within the biomedical field, REEs have been primarily used as diagnostic and imaging agents. Thus, low-molecular-weight gadolinium (Gd) complexes have been established as contrast agents in MRI imaging due to their high thermodynamic and kinetic stability as well as unique magnetic properties that enable the enhancement of the relaxation rate of water protons in tissues [5]. Europium (Eu) and terbium (Tb) chelates can be successfully exploited in optical imaging, which can be coupled with MRI in order to provide both the high spatial resolution specific for MRI and the high sensitivity that characterizes optical imaging [6]. Various types of nanoparticles doped with REEs have been designed for multimodal imaging and theranostic procedures that combine luminescence, photothermal effects, and radiation sensitivity [7].

However, the current review focuses specifically on the direct anticancer activity of REEs rather than their roles in imaging and diagnostics, the latter being extensively covered elsewhere [8].

Anticancer research is currently a top priority subject matter since cancer remains a leading cause of morbidity and mortality worldwide, mainly due to the fact that conventional therapies, such as chemotherapy and radiotherapy, are often limited by systemic toxicity, lack of selectivity, and the development of drug resistance [9]. These challenges are continuously triggering new searches for improved therapeutic approaches; these include the design and development of metal-based agents that can either combine or replace current therapeutic standards by employing unique biological targets or mechanisms of action. Metal-based chemotherapy has played an essential role in oncology ever since the discovery of cisplatin, which was the first metal-based anticancer drug introduced in clinical use. Despite its long-term use, notable side effects, and frequent drug resistance, cisplatin remains today one of the most widely used anticancer agents due to its high efficacy, serving as a reference for the development of other metal-based anticancer drugs [10]. Cumulative toxicity and drug resistance have triggered the exploration of other platinum-based drugs, which, unfortunately, have lacked superior anticancer effects. Moreover, they exhibited similar unfavorable pharmacotoxicological profiles with very few reaching the level of clinical trial and even fewer entering clinical use [11]. Alternative metal ions have also been employed in anticancer research in an effort to engage fundamentally different mechanisms of action [12]; in this context, rare-earth metals differ significantly from transition metals through their weak ligand field effect, coordination chemistry, and poor redox activity within the physiological environment that may enable particular biological behavior.

The two main types of rare earth metal-based anticancer agents are molecular complexes and nanostructures. The molecular complexes are coordination compounds between rare earth elements and biologically active ligands, preferably containing oxygen and nitrogen, such as amides, carboxylates, and various heterocycles. Within these compounds, the bonding results strictly from electrostatic contact and, thus, the coordination geometry is controlled by steric variables [13]. Such coordination compounds revealed selective cytotoxicity against tumor cells by inducing apoptosis via mitochondrial dysfunction and cell cycle arrest. Therefore, evidence suggests that the REE coordination chemistry can be tailored to alter critical cancerous pathways such as ROS generation, membrane interaction, and intracellular signaling [14]. The second choice, nanostructures, further expands the range of potential functional anticancer strategies by integrating various organic and inorganic compounds into multifunctional platforms. These platforms are able to both passively accumulate in tumor tissues through the enhanced permeability and retention (EPR) effect and actively target specific receptors through surface-modifying ligands. Additionally, they may benefit from reduced systemic toxicity due to their ability to selectively target cancer cells. Their potential to carry large amounts of anticancer agents enables them to overcome multidrug resistance mechanisms and to simultaneously target multiple pathways involved in cancer development and progression [15]. Although the vast majority of research focused on the diagnostic or theranostic properties of metal-based nanoparticles, such as imaging-guided drug delivery, evidence is currently accumulating on the direct anticancer effects of REE-based nanomaterials. The investigation of REE nanoparticles highlighted their potential antioxidant activity as well as their interference with regulatory signaling pathways that may change the therapeutic outcomes in malignant pathologies.

Given that the imaging and diagnostic applications of REE-based compounds/nanoparticles, including MRI contrast agents and PET/SPECT radiotracers, have been comprehensively reviewed elsewhere [16,17], they are excluded from the scope of this article. This review instead discusses rare-earth molecular complexes and nanostructures that exhibit direct anticancer activity, such as intrinsic cytotoxicity, radiosensitization, and modulation of reactive oxygen species. By including scandium and yttrium alongside lanthanides, we hope to provide a unified perspective on how REE chemistry, from well-defined coordination compounds to multifunctional nanomaterials, can be exploited for the development of future anticancer agents.

## 2. Methodology of Research

**Figure 1:**
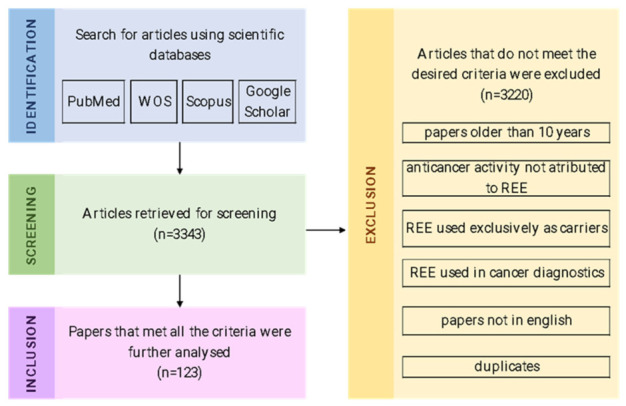
Study selection process.

This article analyzed the papers published in the period between 2016 and February 2026 that describe the anticancer effects of REEs. We searched several databases i.e., Web of Science, Scopus, Pubmed and Google Scholar, using “Terbium”, “Thulium”, “Yttrium”, “Scandium”, “Ytterbium”, “Cerium”, “Erbium”, “Dysprosium”, “Europium”, “Gadolinium”, “Holmium”, “Lanthanum”, “Lutetium”, “Neodymium”, “Praseodymium”, “Promethium”, “Samarium”, “Rare Earth elements”, as well as “anticancer”, “antitumour” and “cytotoxic” as keywords. Three experienced researchers verified all the results and applied the inclusion and exclusion criteria. All in vitro, in vivo, and clinical studies were included in the analysis, along with review articles. We excluded all the articles older than 10 years, studies where REEs were used exclusively as anticancer drug carriers, papers where REEs were used in diagnostic and imaging purposes, and articles where the anticancer effect could not be attributed to REEs. Finally, papers that met all the above-mentioned criteria were analyzed (Figure 1).

## 3. Terbium

Terbium (Tb) is a lanthanide element (Z = 65) that predominantly exists in the +3-oxidation state (Tb^3+^) (Table 2). It exhibits 4f–4f electronic transitions that are able to trigger sharp green luminescence emissions around 545 nm, as well as long excited-state lifetimes within the milliseconds range; this behavior makes this element highly suitable for time-resolved fluorescence bioimaging and luminescence assays [8]. Tb^3+^ is able to form stable coordination complexes with multidentate ligands containing nitrogen and/or oxygen, generally exhibiting 7–9 as the coordination number [18]. It also shows high thermodynamic stability, characterized by formation constants often in the range of 18–25, depending on the ligand environment [19,20]. These complexes display strong binding affinity to biomolecules such as serum albumin, where the binding constants reach values of 10^5^–10^6^ M^−1^ and the interaction occurs preferentially at site III of albumin (subdomain IB [21,22,23]. Similarly, Tb complexes interact with DNA, with binding constants in the range of 10^4^–10^6^ M^−1^ [24], and can mediate DNA cleavage [25]. These chemical and thermodynamic features, combined with relevant anticancer, antimicrobial, and targeted delivery potential, make terbium-based complexes highly promising candidates as diagnostic, therapeutic, and drug-delivery agents.

Terbium was used as a nanoscintillator in X-ray-induced photodynamic therapy, being included in ultra-small gadolinium-based nanoparticles as a doping element; the activation of the photosensitizer molecule led to the generation of singlet oxygen and radical species in U-251 MG human glioblastoma cells. Terbium replaced gadolinium as a doping element in the gadolinium-based nanoparticles, thus allowing the X-ray photon energy transfer from terbium to the photosensitizer molecule, which in turn induced an increased cell growth arrest [27]. Two complexes containing lanthanum and terbium, respectively, as well as the 2,20-bipyridine ligand, were synthesized by Aramesh-Boroujeni et al. as antibacterial and antitumor agents [23]. The study showed that both the terbium and lanthanum complexes can strongly bind to bovine serum albumin (BSA) within the physiological environment through a static quenching mechanism, mainly via hydrogen bonding and van der Waals interactions. Site III (subdomain IB) was identified in BSA as the binding site through multispectroscopic experiments, Förster Resonance Energy Transfer (FRET) analysis, and molecular simulations, with terbium complexes exhibiting higher binding affinity compared to the lanthanum complexes. In addition to protein binding, both complexes revealed antibacterial, antifungal and antitumor activities. Their antitumor activity was assessed in A-549 (lung cancer) and MCF-7 (breast cancer) cells and also in normal human fibroblasts (HFB) for selectivity assessment. The comparison with conventional anticancer drugs such as 5-fluorouracil and methotrexate emphasizes that the lanthanide complexes exhibit comparable anticancer activity combined with lower cytotoxicity in normal cells.

The biological potential of a terbium–2,9-dimethyl-1,10-phenanthroline (Tb–Me_2_Phen) complex was investigated in terms of anticancer, antimicrobial, and DNA-binding properties [28]. By means of a combination of physicochemical techniques, competitive binding experiments, molecular docking, and gel electrophoresis, the complex was revealed to bind strongly to both DNA and BSA, mainly through groove-binding interactions; site III in BSA was identified as the binding site. The Tb complex also exhibited the ability to cleave DNA as well as to induce significant antibacterial and anticancer effects against human cancer cell lines. Moreover, lipid- and starch-based nanoencapsulation further enhanced its anticancer efficacy.

Conversely, five novel metal–organic complexes containing the 5-aminopyridine-2-carboxylic acid ligand were synthesized and assessed, with three of these containing terbium, erbium, and ytterbium ions, respectively, which exhibit slow magnetic relaxation and luminescent properties. The other two are transition-metal multidimensional coordination polymers based on cobalt and copper, respectively, which show tunable structural and emissive behavior due to the aromaticity and flexible coordination modes of the ligand. Their biological activity was assessed in B16-F10 murine melanoma, Hep-G2 human hepatocarcinoma, and HT29 human colon carcinoma cells. The results indicated selective cytotoxicity against all three cell lines, with the cobalt complex exerting the highest activity, while the Tb complex was revealed to be inactive. The choice of the lanthanide element significantly influenced the complex’s biological activity, thus suggesting that the tuning of the metal element allows the modulation of the cytotoxic properties of the complex while simultaneously retaining its multifunctional profile [29].

In another study, Tb(III) complexes were synthesized using Tb(NO_3_)_3_·6H_2_O and four different aroylhydrazine ligands; their structural characterization indicated that each ligand binds coordinatively with Tb(III), resulting in a 1:1 metal-to-ligand ratio binuclear complex with the coordination number 9. These complexes can interfere with the calf thymus DNA with binding constants in the range of 10^6^–10^7^ M^−1^, which indicates their potential as anticancer agents. In addition, all complexes display strong antioxidant activity toward hydroxyl radicals (•OH) and superoxide anions (O_2_^−•^); complexes containing phenolic hydroxyl groups are more effective at scavenging •OH, while those with N-heteroaromatic substituents exhibit stronger activity against O_2_^−•^. Moreover, one particular complex showed lower superoxide scavenging activity, thus indicating that the mechanisms of radical scavenging differ between •OH and O_2_^−•^ and warrant further investigation [30]. Brayshaw et al. revealed that terbium can competitively replace calcium in cadherins, which are key calcium-binding proteins involved in cell adhesion and, subsequently, in embryo development, tissue homeostasis, and tumor metastasis [31]. Cadherins require the binding of Ca^2+^ ions at all 12 active sites in order to achieve their functional conformation; therefore, the loss of Ca^2+^-binding, even at a single site, may disrupt cadherin-mediated cell adhesion. Consequently, the replacement of calcium with terbium disrupts the cadherin structure, increases ectodomain flexibility, abolishes cell-adhesive function, and makes cadherins more susceptible to proteolysis. Such findings emphasize that the lanthanide–calcium substitution alters cadherin dynamics and function and may provide an efficient anticancer effect. The authors also highlighted the fact that all lanthanides are able to chemically mimic calcium ions and are therefore suitable to interact with the Ca^2+^-binding pockets of classical cadherins.

Recent studies have demonstrated that coordination nanocrystals integrating X-ray scintillators and photosensitizers can significantly enhance the efficiency of X-ray-induced photodynamic therapy. For example, terbium–rose bengal coordination nanocrystals synthesized via solvothermal methods were designed to reduce photon energy dissipation between Tb^3+^ and the photosensitizer [32]. Due to their ultrasmall crystalline structure (~7 nm) and efficient scintillation–radiosensitization coupling, these nanocrystals generated singlet oxygen and hydroxyl radicals under low-dose X-ray irradiation, thus producing substantially higher ROS levels than the free photosensitizers or the inorganic nanoparticle used as a control. The nanocrystals exhibited low intrinsic cytotoxicity and efficient cellular internalization, and induced a pronounced DNA damage, which resulted in tumor cell death.

Schiff base ligands represent interesting, versatile platforms for lanthanide-based photodynamic agents. A newly synthesized Schiff base resulted from 4-methoxysalicylaldehyde, and phenyl-hydrazono-acetaldehyde was used to prepare optically active samarium, terbium, and gadolinium complexes via a one-pot reaction [33]. The resulting complexes exhibited strong UV absorption within the 280–390 nm range, intense phosphorescence extending into the near-infrared region (up to 830 nm), low charge-transfer energies (~2.6 eV), and high molar extinction coefficients, which collectively enable efficient triplet–triplet energy transfer and singlet oxygen generation. Due to their thermal stability and favorable photophysical properties, such complexes show potential as photodynamic therapy agents able to achieve apoptosis-mediated cancer cell ablation.

Green synthesis strategies have recently been explored for the preparation of inner transition metal-based nanoparticles. Biocompatible terbium oxide nanoparticles with well-defined crystalline and optical properties were biosynthesized using the fungus *Fusarium oxysporum* [34]. The resulting nanoparticles, showing diameters of around 10 nm, revealed selective cytotoxic effects against two osteosarcoma cell lines (MG-63 and Saos-2) with an IC_50_ value of 0.102 µg/mL; in primary osteoblasts, when applied in lower concentrations, the nanoparticles lacked any toxic effect. Mechanistic investigations revealed that the recorded cytotoxicity was mainly exerted through increased intracellular ROS generation and subsequent oxidative stress, DNA damage, nuclear fragmentation, and the activation of apoptotic pathways. The cells responded in a dose-dependent manner with a threshold behavior, thus emphasizing the role of oxidative stress-induced apoptosis in the anticancer activity of Tb_2_O_3_ nanoparticles.

**Figure 2:**
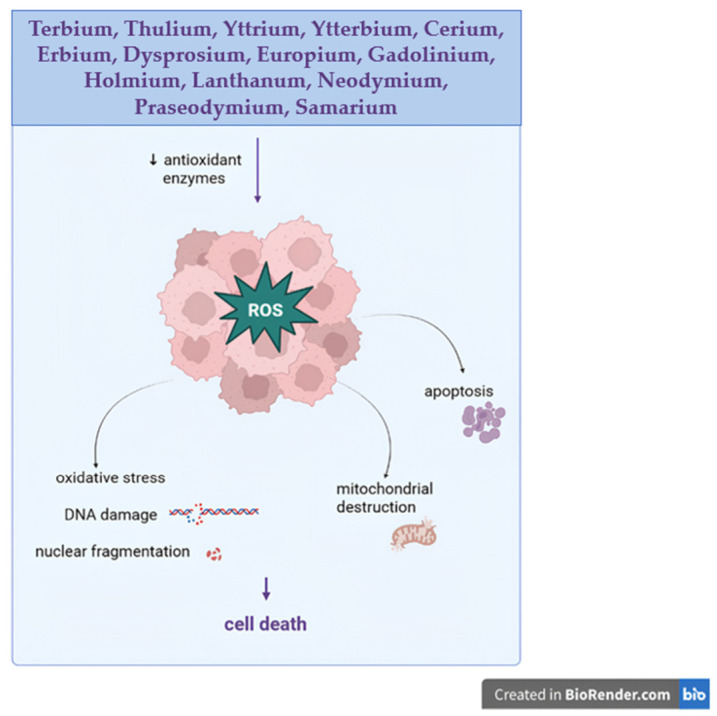
The effects of terbium, thulium, yttrium, ytterbium, cerium, erbium, dysprosium, europium, gadolinium, holmium, lanthanum, neodymium, praseodymium, and samarium on ROS production in cancerous cells (Created in BioRender, 30 March 2026).

Tb exhibits pharmacokinetic, biocompatibility, and long-term properties that emerge from its high charge density, high affinity for oxygen-donor ligands such as phosphates and carboxylates, and a close ionic similarity with calcium ions that enable its interference with calcium-dependent biological processes. Therefore, one may state that Tb competes with calcium in terms of binding to specific sites in proteins such as calmodulin, ion channels, and Ca^2+^-ATPases, thus subsequently altering the intracellular signaling, enzyme inhibition, and neurotransmitter regulation [35,36,37,38]. However, unlike calcium, Tb forms more stable complexes with the biological ligands, which are therefore prone to prolonged remanence periods in the body and cumulative effects. Following systemic administration, Tb exhibits fast plasma clearance but is preferentially redistributed to liver and bone tissues where, due to its affinity for phosphate ligands, has the ability to incorporate in hydroxyapatite; in this form, Tb may cumulate in such tissues that become long-term deposits from which Tb ions can be released slowly into the plasma, thus showing an extended biological half-life [39,40]. Such cumulative effects in bones have been associated with alterations in bone mineral density as a result of chronic exposure to the environment [41]; conversely, this bone preference can be exploited in targeting bone pathologies such as bone metastasis in certain malignancies [42]. At the cellular level, Tb has the ability to induce toxicity via several pathways, such as oxidative stress, membrane disruption, and genotoxicity. Although under physiological conditions, Tb does not engage in redox reactions, it is capable of interfering with the mitochondrial function and calcium homeostasis, thus leading to increased levels of ROS, lipid peroxidation, and DNA damage (Figure 2) [43,44]. Additionally, Tb ions can induce transcriptomic and signaling alterations that include the regulation of genes involved in processes like apoptosis, inflammation, and responses to cellular stress, therefore, being able to trigger chronic pathologies [45,46]. Tb may also induce long-term neurotoxicity by altering neurotransmitters and enzyme activity as a result of chronic exposure. Thus, Tb phosphate was able to disrupt the dopaminergic and serotonergic pathways by decreasing the levels of dopamine, serotonin, as well as their precursors and metabolites, downregulating tyrosine hydroxylase, the rate-limiting enzyme for dopamine synthesis, and reducing levels of monoamine oxidase that catabolizes both dopamine and serotonin [47]. Terbium metabolic clearance is strongly limited by its own coordination chemistry that compels Tb ions to rapidly form stable complexes with plasma proteins and extracellular matrices, subsequently hindering its renal elimination and reorienting distribution toward the liver and bones, as previously mentioned. Tb is eliminated from these tissues through either the hepatobiliary pathways or the bone remodeling processes. This behavior can be, however, significantly adjusted through chemical and nanotechnological approaches; thus, Tb ions chelation by using macrocyclic ligands such as 1,4,7,10-tetraazacyclododecane-1,4,7,10-tetraacetic acid (DOTA) results in highly stable complexes that are able to avoid transchelation with biologically active ligands and provide optimized renal or hepatobiliary clearance [48,49]. Alternatively, Tb nanoparticles can be functionalized with PEG and amino acids that induce improved stability in colloidal systems, reduce their nonspecific binding, and therefore improve biocompatibility [50,51]. Nonetheless, even the use of such systems cannot be regarded as completely safe for therapeutic application in humans due to the fact that low levels of oxidative stress or mild chronic inflammation under prolonged exposure have been reported [52].

## 4. Thulium

Thulium is a rare-earth metal with no known biological function. Due to its high atomic number (Z = 69, higher than gadolinium) (Table 2) and its paramagnetic properties, it shows potential for applications in computer tomography and magnetic resonance imaging. Thulium is mainly used in laser technologies, including medical lasers, being increasingly applied since 2005 in the treatment of benign prostatic obstruction. Moreover, thulium-based and thulium-doped nanoparticles are gaining attention in biophotonics for applications in bio-imaging, nanothermometry, and hyperthermia-based diagnosis and therapy [53]. Thulium oxide revealed a strong potential to act as a radiosensitizer in metastatic skin squamous cell carcinoma (cSCC) when used with or without carboplatin systemic treatment [54]. Initially considered for imaging purposes [53], thulium oxide nanoparticles with diameters of 40–50 nm were tested in human patient-derived cell lines of metastatic cSCC, where cellular uptake did not impact the short-term cell survival. When exposed to X-ray radiation in the presence of thulium oxide nanoparticles, cell sensitivity increased by a factor of 1.24, with or without low-dose carboplatin pre-sensitization.

**Figure 3:**
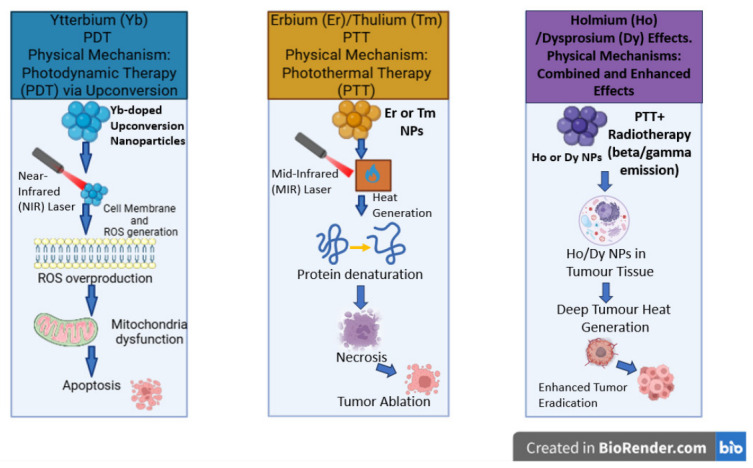
Anticancer effects of REEs due to the physical properties (photothermal/photodynamic) of ytterbium, erbium, thulium, holmium, and dysprosium (Created in BioRender, 31 March 2026).

Smaller (12 nm) thulium oxide (Tm_2_O_3_) nanoparticles were prepared through thermal decomposition and coated by cancer–thylakoid hybrid membranes for the purpose of dual-stage light-guided tumor phototherapy (660 nm and 808 nm), where Tm_2_O_3_ acts as a type II photosensitizer. Due to its NIR energy levels and the long lifetime of the Tm ^3^H_4_ state, Tm_2_O_3_ enables efficient singlet oxygen and ROS generation for photodynamic therapy. The thylakoid membrane provides self-supplied oxygen by catalyzing the decomposition of hydrogen peroxide to oxygen. Thus, it acts as a natural photosensitizer while cancer cell membrane camouflage enhances tumor targeting and immune system evasion. In vitro, the nanoparticles preferentially accumulated in 4T1 (breast cancer) cells rather than fibroblasts, while in vivo, the nanoparticulate system has the ability to effectively eliminate breast tumors in mice through the application of photodynamic and photothermal therapies with synergistic effects [55]. A similar approach consisted of the preparation of Tm_2_O_3_ nanoparticles through thermal decomposition, followed by their coating with polyacrylic acid in order to increase their aqueous solubility [56]. The nanoparticles showed the ability to trigger reactive oxygen species generation. When tested in vitro, they induced low cytotoxicity toward normal L929 cells and limited toxicity toward 4T1 cancer cells, thus revealing significant biocompatibility. Following light irradiation, they induced strong phototoxicity in cancer cells, with a significantly lower IC_50_ under illumination compared to dark conditions, thus confirming effective ROS-mediated photodynamic activity. In vivo, photodynamic therapy (PDT) treatment significantly inhibited tumor growth, with superior efficacy recorded under both NIR laser and non-laser broadband light sources while lacking noticeable systemic toxicity or organ damage (Figure 3).

A thulium (III) terpyridine complex embedding a light-harvesting antenna with strong absorption in the therapeutic window (>590 nm) was synthesized and evaluated as a potential photosensitizer for photodynamic therapy. Following irradiation, the complex exhibited a pronounced phototoxicity against cancer cells due to the generation of reactive oxygen species as the main mechanism of action. Cell viability assays revealed a tenfold reduction in the IC_50_ value under light compared to dark conditions, thus emphasizing its strong potential for PDT anticancer therapy [57]. Thulium oxide nanoparticles also show strong potential as cancer-selective radiosensitizers, in particular against radioresistant tumors such as glioblastoma [58]. In vitro studies revealed their preferential uptake by 9LGS gliosarcoma cells compared to MDCK noncancerous cells, with minimal long-term toxicity in healthy cells. In cancer cells, excessive nanoparticle accumulation led to vacuolization, necrosis, and late-stage apoptosis that may stand as the underlying mechanism of their tumor-selective toxicity. These findings contradict those of Mishchenko et al., who obtained upconversion nanoparticles (UCNPs) co-doped with ytterbium and thulium ions that lacked detectable cytotoxicity toward glioma cells [59]. However, at high concentrations, they induced toxic effects in primary neuronal cultures such as reduced cell viability and altered neuron–glia network activity (Figure 2). Consequently, the authors concluded that the use of UCNPs should be subject to careful evaluation in terms of accumulation and clearance in both tumor and healthy brain cells, as well as surface tuning strategies to mitigate neurotoxicity.

The metabolic clearance, biocompatibility, and long-term toxicity of nanomaterials containing thulium or thulium compounds, such as thulium oxide, remain only partially identified. Current evidence suggests that the biocompatibility of thulium oxide nanoparticles depends on their environment; thus, in non-malignant cells and moderate concentrations, the in vitro tests revealed their low cytotoxicity, while in certain cancer cells, their uptake and cytotoxic effects were significantly increased due to well-known differences between normal and cancer cells in terms of particle internalization via endocytosis and intracellular metabolism [58]. Simultaneously, studies conducted on rare-earth upconversion nanoparticles containing thulium as dopant revealed that their physicochemical parameters, such as the particle size, surface charge, and functionalization, as well as aggregation, are able to orientate the formation of the protein corona, opsonization, cellular uptake, and subsequent biodistribution [60]. Inside the body, nanoparticles interact with plasma proteins and form a dynamic corona that plays an essential role in immune recognition, thus influencing their clearance through mononuclear phagocytosis in the liver and spleen [60]. Although not yet specifically reported in the literature, thulium nanoparticles are expected to undergo similar metabolic pathways as other inorganic nanomaterials; therefore, particles larger than 5–6 nm are incapable of overcoming the renal filtration threshold and are therefore prone to accumulate in reticuloendothelial tissues. As a result, thulium nanoparticles may exhibit longer circulation time and the potential to induce signs of chronic exposure [61,62].

Surface tuning through PEGylation or other hydrophilic coatings may hinder protein adsorption and minimize their aggregation tendency and thus provide a partial escape from immune recognition, which, in turn, may contribute to altered biodistribution and clearance kinetics. However, although the pharmacokinetic parameters change, these modulations of the nanoparticles ‘structure do not enable the degradation of the rare-earth core, which remains too stable to undergo physiological metabolism. Subsequently, the issue of long-term toxicity is not necessarily a consequence of acute toxicity but of bioaccumulation and remanence within the body that may trigger a state of persistent low-grade inflammation, oxidative stress, and organ toxicity. Although the literature is rather scarce in terms of in vivo data regarding thulium, one may extrapolate the results reported for other rare-earth metals that show a chronic retention in organs such as the liver or the spleen, which may lead to subtle but cumulative biological effects that may be missed in short-term studies [63,64]. Therefore, although in controlled in vitro studies the nanoparticles containing thulium displayed promising biocompatibility data, the lack of broader pharmacokinetic studies stands as a critical gap. In order to address this gap, future in vivo investigations are required to clarify the fate of the nanoparticles over an extended time period, including their potential effects at the molecular, cellular, and tissue levels.

## 5. Yttrium

Considered a pseudo-lanthanide, yttrium (Y) is actually a transition metal (Z = 39) whose chemistry resembles that of heavy lanthanides due to its stable +3 oxidation state and comparable ionic radius (Table 2). The [Kr]4d^0^ configuration produces diamagnetic, colorless Y(III) complexes with coordination numbers between 6 and 9.

**Figure 4:**
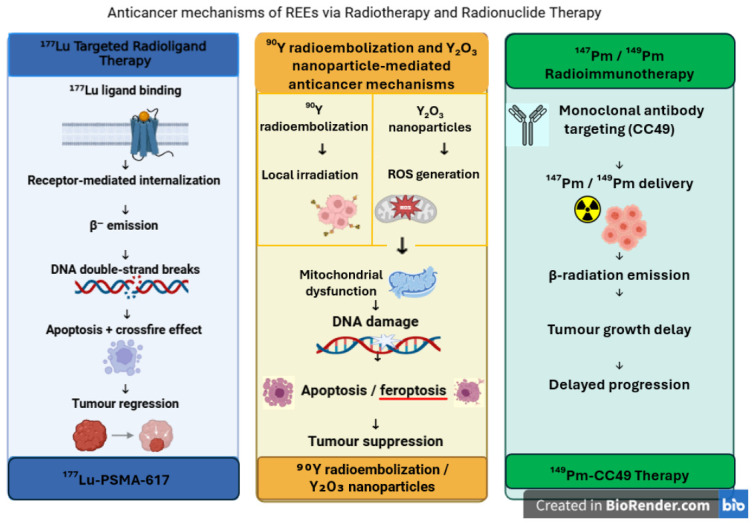
Anticancer mechanisms of REEs via radiotherapy and radionuclide therapy. The figure schematically illustrates targeted radioligand therapy for lutetium, radioembolization, and Y_2_O_3_ nanoparticle-related pathways for yttrium, and monoclonal antibody-guided radioimmunotherapy for promethium (Created in BioRender, 31 March 2026).

Yttrium-based metal–organic frameworks have multifunctional properties and applications, such as luminescence [65] and bioimaging [66], adsorption [67,68], environmental [69], and catalytic [70]. In addition, Y-compounds can act as artificial nucleases by mimicking enzymatic behavior [71,72,73,74]. ^90^Y is one of the most frequently used radioisotopes in radiotherapy and radioembolization to treat hepatocellular carcinoma and metastatic liver cancer (Figure 4) [75]. Yttrium oxide (Y_2_O_3_) induces low toxicity, high thermal stability, and suitable optical properties that make it useful in advanced materials and biomaterials [76].

Cubic yttrium oxide (Y_2_O_3_) nanoparticles displaying an average size of 14 nm and high colloidal stability were assessed against human triple-negative breast cancer (MDA-MB-231) as well as normal cells, namely dermal fibroblast (HDF) and retinal pigment epithelial cells (REP1) [77]. The MTT assay proved that yttrium oxide nanoparticles presented a strong effect on the MDA-MB-321 cell line, but only minimal toxicity against HDF and REP1 cells. In cancer cells, the IC_50_ value was approximately 74 µg/mL, while in normal cells, the respective value was above 100 µg/mL, which suggests moderate selectivity towards malignant cells. Regarding the molecular mechanism, reactive oxygen species (ROS) proved to have a significant role in anticancer activity. The yttrium oxide nanoparticles led to a significant increase in ROS at the intracellular level in breast cancer cells (Figure 2); in opposition, no elevation of ROS was observed in retinal pigment epithelial cells. In breast cancer and dermal fibroblast cells, treatment with yttrium nanoparticles was followed by the occurrence of oxidative stress indicators. The nanoparticles caused a significant increase in malondialdehyde (MDA), reduced glutathione (GSH), and catalase activity (CAT) in cancer cells, which could all be indicative of oxidative stress induction, accompanied by antioxidant response. On the other hand, GSH and MDA also displayed increased levels in HDF cells, while CAT remained unchanged, thus suggesting a steady redox equilibrium in normal cells. Collectively, these results indicate that Y_2_O_3_ nanoparticles preferentially induced an oxidative stress-dependent cytotoxicity in triple-negative breast cancer cells and exerted negligible effects in normal cells, thus revealing their potential as an excellent nanomaterial for anticancer applications. The mechanistic studies revealed that exposure to Y_2_O_3_ nanoparticles dramatically improved both early and late apoptosis in MDA-MB-231 cells. The evaluation of gene expression revealed elevated CASP3 and CASP8, both known pro-apoptotic markers associated with ferroptosis gene heme oxygenase-1 (HO-1) and lower expression of the anti-apoptotic gene Bcl2. These alterations suggest that the nanoparticles are able to induce programmed cell death by activating certain apoptotic pathways and might also trigger ferroptotic mechanisms. In summary, it can be noted that the Y_2_O_3_ nanoparticles demonstrated selective anticancer activity by multiple approaches, such as DNA damage by ROS action, and also apoptosis and ferroptosis induction.

Using *Forsythiae fructus* extract as a reduction agent in an environmentally friendly synthesis method, yttrium oxide (Y_2_O_3_) nanoparticles were obtained. The nanoparticles presented an average size of 11 nm and a flake-like morphology [78]. The newly synthesized nanoparticles were assessed in terms of cytotoxic effects against normal kidney epithelial cells (MDCK) and human renal carcinoma cells (Caki-2) by using concentrations ranging from 1 µg/mL to 1 mg/mL. In Caki-2 cells, a dose-dependent cytotoxic effect was observed after exposure to nanoparticles, marked by a 40% apoptosis rate at concentrations of 1 mg/mL (the highest tested concentration). On the contrary, in MDCK cells, no substantial cytotoxicity was noted, thus proving selective toxicity against cancerous cells.

Across three studies on epidermoid skin [79], hepatic [80], and pancreatic [81] cancers, commercially available Y_2_O_3_ nanoparticles were tested. The nanoparticles displaying a unified spherical morphology were of high purity and stability, and also had an average particle size of approximatively 14 nm.

In the first study, the anticancer potential of Y_2_O_3_ was investigated against human epidermoid skin cancer (A-431) cells by comparison to normal human skin fibroblasts (HSF) [79] by using concentrations ranging between 0.1 and 1000 µg/mL. Using cell viability assays, the authors reported that the Y_2_O_3_ nanoparticles were able to induce a strong, concentration-dependent reduction in A-431 cancer cell viability after 72 h, with an IC_50_ around 30 µg/mL. Conversely, HSF cells showed minimal changes in viability for low to moderate concentrations, with significant cell death reported only at the highest tested dose (1000 µg/mL) and exhibiting an IC_50_ value of about 202.5 µg/mL.

The Y_2_O_3_ nanoparticles were also tested in human hepatocellular carcinoma (Hep-G2) cells [80] by using a wide concentration range (0.2–2000 µg/mL); a clear dose-dependent decrease in cell viability was recorded. The strong anticancer activity was further supported by a low IC_50_ value of 13.15 µg/mL. Furthermore, the Hep-G2 cells treated with the nanoparticles at the IC_50_ concentration produced a significantly increased level of intracellular ROS, thus indicating the important role played by oxidative stress in the cytotoxic mechanism.

Lastly, the Y_2_O_3_ nanoparticles were tested against human pancreatic cancer (PANC-1) cells and normal human skin fibroblasts (HSF) for comparative analysis [81]. Following 72 h of treatment, a significant, dose-dependent inhibition of cancer cell proliferation was recorded. For PANC-1 cells, the IC_50_ had a value of 31.06 µg/mL in comparison to 319.21 µg/mL for HSF cells. This selective toxicity was further supported by a high selectivity index, while the apoptosis assay revealed increased levels of apoptotic and necrotic cells. Such results confirm that Y_2_O_3_ nanoparticles were able to successfully inhibit PANC-1 cell proliferation, while having minimal harmful effects in HSF cells.

The anticancer effects exerted by Y_2_O_3_ in A-431, Hep-G2, and PANC-1 cells, respectively, result through the mitochondria-dependent intrinsic apoptotic pathway, which is mediated by oxidative stress. In all cancer cells, the treatment led to an increase in intracellular ROS production, reduced antioxidant activity, and enhanced lipid peroxidation. Additionally, it caused significant DNA damage, finally leading to the loss of mitochondrial membrane potential. In A-431 cells, apoptosis was strongly correlated with ROS accumulation and a pronounced loss of mitochondrial membrane potential coupled with p53 activation. The mitochondrial damage in Hep-G2 cells demonstrated the essential role of p53 and mitochondrial ND3, which exhibited massively increased values accompanied by the downregulation of Bcl-2, together confirming the activation of the intrinsic apoptotic pathway and the role of the mitochondrial genome. Using flow cytometry, it was further confirmed that ROS generation and DNA fragmentation in PANC-1 cells increased following treatment with Y_2_O_3_ nanoparticles, whereas the expressions of p53, ND3, and Bcl-2 were regulated in a coordinated manner. Although the intensity of the cytotoxic activity varied, a uniform molecular mechanism was reported in all three cancer cell lines: ROS super-production, loss of mitochondrial membrane potential, overexpression of p53 and ND3, as well as Bcl-2 reduction. The findings reveal that Y_2_O_3_ nanoparticles were able to effectively induce intrinsic apoptosis in various cancer cells.

Jahani et al. [74] studied the biological effect and therapeutic potential of yttrium (III) complex with 2,9-dimethyl-1,10-phenanthroline as ligands. The objective in this study was to highlight its anticancer/antibacterial activities, but also its protein affinity and DNA binding. Multiple spectrophotometric techniques and computational investigations demonstrated that a strong bond forms between the yttrium complex and both fish DNA and bovine serum albumin (BSA), respectively, through a static binding mechanism. The ability and potency of the complex to cleave DNA were also investigated. Two nanocarriers, based on lipid and starch, respectively, were developed and tested in order to increase the biological activity of the yttrium complex. The MTT assay in MCF-7 breast cancer cells and A-549 alveolar basal epithelial cells using concentrations between 0.5 and 64 µg/mL revealed a dose-dependent anticancer activity for all tested compounds, with the nano-formulations exhibiting higher toxicity than the free complex. The IC_50_ values of the free yttrium complex were 4.21 µg/mL and 8.86 µg/mL in MCF-7 and A-549 cells, respectively, thus showing a moderate toxicity. For the lipid nano-formulation, the lowest values of IC_50_ were registered as follows: 1.69 µg/mL in MCF-7 cells and 3.94 µg/mL in A-549 cells. Intermediate activity was displayed by the starch nano-formulation with IC_50_ values of 3.38 µg/mL (MCF-7) and 7.16 µg/mL (A-549). Taken together, the results show that the yttrium (III) complex and the two nano-formulations are worthy of further research as anticancer and antimicrobial agents with promising biochemical properties.

Jomma et al. [82] investigated the toxicokinetic behavior of REE oxides (REE: Y, Nd, Pr, Ce) on Sprague–Dawley male rats. After intravenous administration of the insoluble oxides at doses of 0.3 mg/kg or 1 mg/kg, the rats were monitored for a period of 21 days. The data were also compared to the toxicokinetics obtained in similar conditions for REE chlorides [83]. The results indicated that an increased dose of REEs could favor accumulation, especially in the case of insoluble REEs (oxides), which, compared to soluble REEs (chlorides), are more slowly eliminated from the body. Y, Pr, Nd, and Ce oxides have a similar profile, decreased urinary elimination, and persistent retention. On the other hand, some specific characteristics were noticed for each element: Y and Nd had a more intense pulmonary accumulation, Ce had strong hepatic retention, and Pr exerted a uniform distribution.

Panyala et al. conducted two studies investigating the effect induced by nano and micro particles of yttrium oxide on Wistar rats. The first study studied acute exposure to orally administered Y_2_O_3_ [84] and the second explored the effects induced by repeated Y_2_O_3_ administration after 28 days [85]. The two studies confirmed systemic absorption, as well as low clearance, with accumulation in the spleen and liver after repeated exposure. In the case of acute exposure, DNA damage and genotoxic effects were noted as more intense for smaller particles. Repeated exposure indicated a dose-dependent lasting genotoxic stress. While for the acute exposure, only limited damage was recorded, and repeated exposure led mainly to hepatic damage and spleen modification.

## 6. Scandium

Scandium (Sc) is a transition metal (Z = 21) with an [Ar]3d^1^4s^2^ electronic configuration, similar to yttrium, often classified as a rare-earth element (Table 2). Sc chemistry is determined by the +3 oxidation state ([Ar]), which leads to stable, colorless compounds with coordination numbers ranging from 3 to 9, but usually preferring six-coordinate complexes [86]. The Sc compounds have demonstrated high versatility for various medical and industrial applications. Recent studies underlined their efficiency as bacteriostatic agents [87], osteogenic stimulators [88], high-performance catalysts [89], and as essential components in biocompatible coatings and implants [90]. Scandium isotopes were used as theranostic agents: ^43/44^Sc was used in PET imaging, while ^47^Sc proved to be a promising candidate as therapeutic radionuclide [91,92].

A series of 12 newly synthesized 3-metallocene complexes with scandium, yttrium, and neodymium were tested for their cytotoxic effects in human breast cancer (MDA-MB-231) and prostate cancer cells (DU145) by Caporale et al. [93]. The most active complex proved to be half-metallocene dichloride with a p-methoxybenzyl-substituted cyclopentadienyl ligand, which exhibited IC_50_ values below 10 µM for the two cancer cell lines. A higher efficiency was noticed for the yttrium compounds, particularly a half-metallocene dichloride incorporating a 3,4-dimethoxybenzyl cyclopentadienyl ligand, and a metallocene monochloride containing two p-methoxybenzyl-substituted cyclopentadienyl ligands, both showing strong cytotoxic activities with IC_50_ values under 5 µM in both cancer cell lines. In contrast, the neodymium structurally identical analogs showed only moderate to low antiproliferative activity, thus indicating a weaker biological response compared to scandium and yttrium. The results mainly indicate the yttrium compounds as the most potent candidates in this series of complexes, followed by scandium compounds with moderate biological effects. Collectively, in addition to the nature of the metal involved, a significant influence is exerted by the coordination mode and the type of ligand substitution.

**Figure 5:**
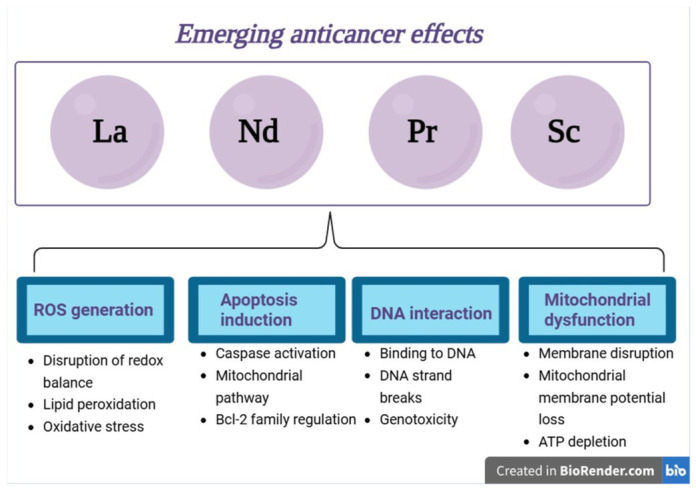
Rare-earth elements (lanthanum, neodymium, praseodymium, and scandium) exhibit emerging anticancer activity based on preclinical evidence. (Created in BioRender, 31 March 2026).

Sc-exopolysaccharides [94] were investigated for their cytotoxic potential in osteosarcoma (MNNG/HOS), melanoma (A375), lung adenocarcinoma (A549), glioblastoma (U251), breast cancer (MDA-MB-231), and colon cancer (Caco-2) cells. Scandium ions revealed a reduced toxic effect, and the exopolysaccharides alone showed moderate cytotoxicity, but the Sc-exopolysaccharide exerted a strong antiproliferative activity against the tested cancer cell lines. The addition of scandium ions led to changes in the exopolysaccharide’s chains that made them interact more easily with the surface of the cancerous cells, thus inducing stronger cytostatic effects instead of direct cytotoxicity (Figure 5). The degree of the exopolysaccharide’s sulfation and metal–ligand proportion altered their antiproliferative efficiency; thus, the highly sulfated complexes, especially in a ratio of 1:2, triggered the most significant reduction in cell proliferation throughout multiple cancer cell types. These findings indicate that the biological action of the exopolysaccharide is increased by the presence of scandium; it acts by interfering with cell adhesion and the pathways of growth factor receptors.

Alshamrani et al. [95] investigated the anticancer activity of scandium coordination complexes against human colorectal carcinoma (HCT116) and human colorectal adenocarcinoma (HT-29). The tested compounds were tetranuclear or dinuclear scandium-calixarene complexes that were stabilized by calix[4] or calix[6] arenes. The tested complexes exhibited low IC_50_ values when tested in vitro and a concentration-dependent behavior. For the low concentration, no toxicity was recorded, while for the higher concentration, only a slight toxicity was observed. Overall, the investigated scandium complexes presented a relatively non-toxic behavior under the tested conditions. Furthermore, the limited data from this study cannot certainly classify these compounds as effective anticancer agents.

The toxicokinetics of scandium were investigated on Sprague–Dawley male rats that were injected with scandium oxide (Sc_2_O_3_) [96]. The blood kinetics indicated a rapid distribution to the lungs and liver, and a slower elimination compared to other soluble rare-earth chlorides [83]. A partial and low excretion rate was noticed, especially in urine, while for fecal excretion, it was noticed that a higher dose led to a diminished excretion. The tissue analysis after 21 days revealed a tendency to accumulation mainly in the lungs (6.6–7.5 µg/g) and in much lower proportions in the spleen (0.9–1.2 µg/g), liver (0.5–0.8 µg/g), kidneys (0.01–0.03 µg/g), and brain (0.002–0.03 µg/g). The highly stable and insoluble Sc_2_O_3_ is trapped by the phagocytic systems in the organs and only slowly eliminated by biliary pathways.

A study of clinical biomonitoring focused on the effects induced by scandium accumulation due to chronic renal dysfunction [97]. Plasma analysis revealed an enhanced level of scandium in patients with impaired renal function compared to healthy controls. The highest amount of Sc was noticed in the predialysis patients, the lowest amount in the healthy patients, and an intermediate Sc level was found in patients undergoing hemodialysis. The study revealed that hemodialysis could not normalize the Sc levels, removing only partially the amount of accumulated element. Moreover, from a clinical perspective, the findings indicate that Sc is not biologically inert and tends to accumulate due to impaired clearance conditions.

## 7. Ytterbium

Ytterbium (Yb) is the penultimate member of the lanthanide series (Z = 70), with the unique ground-state configuration [Xe]4f^14^6s^2^ that, besides the standard +3 oxidation, can also exist in a relatively stable +2 oxidation state (Table 2). Yb^3+^ binds with nitrogen and oxygen ligands, forming stable complexes with high coordination numbers (frequently 6–9) [98], but also inorganic compounds like oxides and halides [99].

Ytterbium compounds are becoming increasingly investigated in biomedical research, mainly at a preclinical level due to their favorable radio-physical and optical properties [100]. Radionuclide ^169^Yb has been used in industrial radiography and explored for medical imaging and brachytherapy applications [101,102]. Ytterbium-containing materials are also investigated as contrast agents [103,104], radiosensitizers [105], and multifunctional nanocarriers [106,107], while Yb-porphyrin complexes [108] have shown promise in cancer theranostics.

Ning et al. prepared a series of beta-fluorinated ytterbium (III)-porphyrin complexes that were tested in HeLa cells for their cytotoxic effect and biocompatibility [109]. Under dark conditions, only a very low toxicity was noticed, with cell viability remaining above 80% for concentrations up to 20 µM. Under light irradiation, most complexes demonstrated negligible photocytotoxicity at 10 µM. On the other hand, the azlactol-derivative complex Yb-5 bearing a trimethyl-*N*-ethylammonium moiety had a significant cytotoxicity induced by light, with an IC_50_ of about 1.0 µM. This phototoxic effect was attributed to a high-energy transfer process that led to ROS formation. The ytterbium complexes proved to be mainly non-toxic under normal conditions but can induce controlled cytotoxicity under irradiation, which enables their use in applications like imaging and photodynamic therapy (Figure 3).

*Couroupita guianensis* leaf extract, YbCl_3_, and 1-butyl 3-methyl imidazolium tetrafluoroborate ionic-liquid (IL) were used in order to obtain Yb_2_O_3_ and ionic-liquid-assisted Yb_2_O_3_ (Yb_2_O_3_-IL) through the hydrothermal method [110]. The synthesis led to the formation of single-phase-containing nanoparticles with diameters ranging between 35–60 nm and 25–55 nm, for Yb_2_O_3_ and Yb_2_O_3_-IL, respectively. The investigated nanoparticles exhibited strong antibacterial, antioxidant, and anti-inflammatory effects; additionally, the MTT assay was used to assess the cytotoxicity of the ytterbium oxide nanoparticles against MCF-7 breast cancer cells. The results showed that both Yb_2_O_3_ and Yb_2_O_3_-IL have a dose-dependent cytotoxic effect in MCF-7 cells, with Yb_2_O_3_-IL exhibiting higher potency as indicated by an almost 90% reduction of cell viability following the application of a 50 µg/mL sample. A similar experiment was conducted by the same research group by using the *Andrographis paniculate* leaf extract as a reducing agent [110]. In this study, the nanoparticle’s size ranged between 35–45 nm for Yb_2_O_3_ and 25–37 nm for Yb_2_O_3_-IL. In a similar manner, the resulting nanoparticles displayed antibacterial effects against both Gram-positive and Gram-negative bacteria, together with strong antioxidant effects and antidiabetic activity. Further in vitro tests revealed significant anticancer effects in MCF-7 cells exerted in a dose-dependent manner; thus, a 83% reduction in cell viability was reported for Yb_2_O_3_ and 93% for IL-Yb_2_O_3_ following the application of 50 µg/mL samples. The authors established that such effects were caused by ROS generation, the release of metal ions, and the formation of free radicals, which subsequently led to the disruption of cell membranes, mitochondria destruction and, finally, cellular death (Figure 2).

A new ytterbium complex was developed using 2,9-dimethyl-1,10-phenanthroline as ligand, with the structural formula Yb(Me_2_Phen)_2_Cl_3_(H_2_O) [111]. The authors investigated the complex’s binding affinity for BSA and fish DNA, as well as its ability to cleave DNA and therefore act as an anticancer and antibacterial agent. The research indicated that the ytterbium complex exhibits significant binding to fish DNA and BSA primarily via hydrophobic interactions. In an effort to enhance its bioactivity, the complex was embedded with two types of nanocarriers, based on starch and lipids, respectively. The cytotoxic effects of the ytterbium complex, as well as its two nano-formulations, were assessed by means of MTT assay in A-549 (lung) and MCF-7 (breast) cancer cells; tests indicated dose-dependent cytotoxic effects across all tested samples, with the lowest IC_50_ values recorded for the lipid nano-formulation, 1.62 µg/mL for MCF-7 and 3.29 µg/mL for A-549 cells, followed by the starch nano-formulation, which exhibited IC_50_ values of 3.36 µg/mL (MCF-7) and 6.80 µg/mL (A-549). For the free ytterbium complex, the IC_50_ values were significantly higher compared to both nanoformulations. The inhibition of cancer cell proliferation was associated with the occurrence of DNA damage, oxidative stress, and disruption of cellular functions. The ytterbium complex also showed antibacterial and antifungal activity due to its enhanced lipophilic character, which induced membrane disruption.

A study conducted by Feng et al. [112] investigated the accumulation tendency of ytterbium following oral administration in various concentrations. Wistar rats were exposed to ytterbium throughout the gestation stage until 5 months of age. It was demonstrated that Yb was widely distributed across the organism and presented retention in the brain and other organs, after pre- and postnatal exposure, in adult rats and their offspring, underlying the long-term accumulation under repeated exposure conditions. In addition, it must be stated that Yb is capable of crossing the placenta barrier.

Adeel et al. [113] conducted a study using a mouse model exposed for a period of 30 days to ytterbium oxide (Yb_2_O_3_) nanoparticles by intranasal inhalation. A significant accumulation was noticed in multiple organs, the highest levels being in the lungs, followed by the liver, kidney, and heart. After repeated exposure to Yb_2_O_3_ nanoparticles, an inflammatory response of the organism and histopathological damage were observed. The most intense point of accumulation in the brain was the olfactory bulb, proving this was the pathway of Yb access to the brain. Interestingly, the study demonstrated that the toxicity, in this case, is due to a mechanism induced by the nanoparticles’ accumulation and not by the intrinsic activity of the Yb^3+^.

## 8. Cerium

Currently, cerium is increasingly targeted by researchers [114] due to its powerful antioxidant, anti-inflammatory, and immunomodulatory effects, as well as its antibacterial and antiviral activity [115].

Cerium (Ce) is a rare-earth metal with the atomic number Z = 58, which exists in two oxidation states: Ce^3+^ and Ce^4+^ (Table 2), being able to make the reversible transition between the two states [116]. The dual property of cerium as an antioxidant and pro-oxidant agent gives it a similar activity to antioxidant enzymes, being able to mimic the functions of superoxide-dismutase, catalase, and other detoxifying enzymes within redox reactions [117]. It is used almost exclusively in the form of cerium oxide nanoparticles (CeO_2_NPs, nanoceria) in fields such as dermatology, neurology, cardiology, and ophthalmology, as well as oncology [114]. Gao et al. reported in 2014 its anticancer effects, but also its safe application in healthy cells; it has been shown that at physiological pH, cerium oxide nanoparticles have an antioxidant effect, protecting tissues from free radicals, while at acidic pH, cerium oxide nanoparticles generate oxidative stress and act as oxidants [118]. Similarly, another study evaluated the dual properties of cerium oxide nanoparticles, where the antioxidant and pro-oxidant effects of cerium in various cancer lines, such as MCF-7 (breast cancer), HeLa (cervical cancer), A549 (lung cancer), and HepG2 (liver cancer), induced apoptosis and inhibited cell proliferation (Figure 2). In addition, in vivo studies conducted in rodents showed a clear tendency of cerium oxide nanoparticles to accumulate in the liver and spleen, as well as slower elimination; however, this hypothesis remains to be investigated further [119]. In another study, Javid H. et al. discuss the activity of cerium oxide nanoparticles in esophageal cancer cells (YM1) and cancer stem cells (CSC-LC). According to their study, cerium oxide nanoparticles were directly dispersed into the culture medium, causing significant decreases in levels of reactive oxygen species (ROS) and malondialdehyde (MDA), while increasing the antioxidant enzymes superoxide dismutase (SOD) and catalase (CAT), as well as thiols and total antioxidant capacity (TAC). The IC_50_ values varied according to the cell line; thus, the IC_50_ value was 758 µM for the YM1 cell line and 968 µM for the CSC-LC cells, thus showing a higher resistance of cancer stem cells to cancer treatment [120]. Instead, Gunasekaran et al. revealed a pro-oxidant effect for cerium oxide nanoparticles applied in lung cancer cells (H460), with a significant increase in ROS at concentrations ranging from 50 to 100 µg/µL, thus indicating the triggering of cancer cell apoptosis [121]. When cerium nanoparticles were decorated on graphene oxide (GO/CeO_2_) sheets to increase their efficacy against breast, brain, and esophageal cancer, the results showed that the strongest anticancer effect was achieved in AMJ13 breast cancer cells. An inhibition of tumor cell growth of 51.04% was reported compared to brain cancer cells (AMGM5), where the percentage of inhibition was only 37.12%. In both cases, a maximum dose of 250 µg/mL was used. In esophageal cancer cells, the results showed a statistically insignificant inhibitory effect, while in normal cells, cerium oxide nanoparticles were reported as safe and biocompatible, almost completely lacking cytotoxic effects [122]. Similarly, in another study regarding cerium nanoparticles fixed on graphene oxide, the additional doping with silver (CeO_2_:Ag/GO) led to a significant increase in anticancer effectiveness. The authors assessed their activity against HT-29 colorectal cancer cells, where a high percentage (>60%) of cancer cell growth inhibition was recorded at doses as low as 50 µg/mL. In addition, these nanoparticles triggered apoptosis by activating the BAX and Caspase-1 genes, simultaneously blocking the tumor protection gene Bcl-2, and showing no toxic effects in normal human cells [123]. To further increase the effectiveness against cancer cells, cerium nanoparticles (CeO_2_ NPs) have been synthesized using the essential oil extracted from pistachio pericarp (*Pistacia vera*) as a stabilizer and assessed in terms of activity against breast cancer (MCF-7) and prostate cancer (LNCaP) cells; the presence of the green stabilizer facilitated the accumulation of a large amount of cerium particles at the tumor target site. The in vitro tests revealed IC_50_ values in the micromolar range for both cell lines. When zoledronic acid was added, a significant decrease in the IC_50_ value was recorded, thus revealing a synergistic effect between cerium nanoparticles and the loaded drug. The study concluded that, although the blank cerium nanoparticles display significant cytotoxic effects, their use in combination with zoledronic acid increases the level of apoptosis [124]. An alternative of harm to cells can be achieved by making oxide nanoparticles with dextran coating. These nanoparticles have molecular weights and branching characteristics like SD1 and SD2. The study looked at how these nanoparticles affect three types of cancer cells: A253, which is a type of salivary gland cancer; SCC-25, which is a type of tongue cancer; and FaDu, which is a type of pharynx cancer. For SD1, it took 315 µg/mL to kill half of the A253 cells and 347 µg/mL to kill half of the FaDu cells. It took more than 1000 µg/mL to kill half of the SCC-25 cells. In contrast SD2 worked better at killing cells. It took 129 µg/mL to kill half of the A253 cells, 292 µg/mL to kill half of the FaDu cells, and 225 µg/mL to kill half of the SCC-25 cells. SD2 worked better than SD1 on all the cancer cells. A253 cells were affected by SD2 followed by SCC-25 and then FaDu cells. This shows that different cancer cells have various levels of sensitivity to these nanoparticles [125]. Also, the combination with radiotherapy strengthens the anticancer effect of cerium oxide nanoparticles in L3.6pl and Panc1 pancreatic cancer lines. A dose of 10 µM of cerium oxide nanoparticles together with a dose of 5 Gy radiotherapy were used, and the results showed that cell death increased by approximately 30–40% for the L3.6pl cell line and 40–50% for the Panc1 cell line, while in hTERT-HPNE normal cells, the cerium oxide nanoparticles induced a protective effect. The in vitro results were confirmed in vivo using athymic mice, where the combined therapy triggered significant increases in the activation of both JNK and caspase 3 while simultaneously revealing synergistic characteristics [126]. Moreover, cerium oxide nanoparticles also have a cytotoxic effect on ovarian cancer cell lines. The results showed that treatment of ovarian cancer cells with cerium oxide nanoparticles (50–100 µM, 48 h) significantly reduced basal ROS levels. In addition, cerium oxide nanoparticles (100 µM) inhibited growth factor-induced migration and invasion (SDF1, HB-EGF, VEGF165, and HGF). In vivo, the intraperitoneal administration of cerium oxide nanoparticles (0.1 mg/kg, every 3 days) to nude mice bearing ovarian xenografts resulted in a significant reduction in tumor growth (*p* < 0.01), which was associated with decreased Ki-67 expression (*p* < 0.001) and reduced tumor angiogenesis, as evidenced by the apoptosis of endothelial cells. Of particular interest is that cerium oxide nanoparticles accumulated in tumor tissue but did not exhibit toxicity in vital organs at the given dose [127]. To observe the safety profile of cerium oxide nanoparticles, a subacute toxicity study was performed that lasted 28 days, and in which no systemic toxic effects were observed in the range of 250mg/loc-1000 mg/loc, indicating a low toxicity. With regard to metabolic clearance, there is no description of the mechanisms, but in biodistribution, there is evidence of the presence of cerium in the liver (191.8 ± 35.1 ng/g), lungs (263.4 ± 30.9 ng/g), spleen (211.2 ± 6.5 ng/g), and kidneys (272.8 ± 20.4 ng/g), which indicates a systemic distribution in the body, thus a certain level of clearance. There is also evidence of good biocompatibility with cerium oxide nanoparticles, as there were no significant tissue responses and the irritation index in implantation was less than 3 [128].

## 9. Erbium

Erbium (Er), with the atomic number Z= 68 and the oxidation state +3 (Table 2), also belongs to the “f” block of the periodic table, and its reactivity is dominated by 4f electrons, which give it distinct magnetic and spectral properties [129].

It is usually used for medical purposes as laser components in dentistry and dermatology [130,131]. In addition, erbium has also revealed anticancer effects, as indicated by Hanan et al., who assessed the viability of highly aggressive U937 lymphoma cancer cells in the presence of commercially available erbium oxide nanoparticles with diameters below 100 nm. Promising results were reported, as the viability of cancer cells significantly decreased (IC_50_ value = 3.20 µg/mL), thus showing cytotoxic effects that were attributed to high ROS generation as well as DNA and mitochondrial membrane potential damage. Both apoptosis and necrosis occurred in a selective manner through the dysregulation of apoptotic (*p53*), anti-apoptotic (*Bcl2*), and mitochondrial *ND3* gene expression [132]. In addition, erbium oxide Er_2_O_3_NP_s_ nanoparticles were also tested in HepG-2 liver tumor cells, where results showed an IC_50_ value of 6.21 µg/mL after 72 h of exposure, indicating a pronounced cytotoxic effect and a marked increase in reactive oxygen species, correlated with DNA fragmentation, thus emphasizing an apoptotic effect in cancer cells [133]. Erbium oxide nanoparticles (Er_2_O_3_) were synthesized using a modified polyol method assisted by the ionic liquid BMIM-PF_6_ (IL-EO) and then compared with Er_2_O_3_ nanoparticles obtained in the absence of the ionic liquid. The in vitro anticancer activity of the two types of nanoparticles was comparatively evaluated using the MCF-7 cell line. The results showed that IL-EO nanoparticles induced a dose-dependent inhibition of cell viability with an IC_50_ value of 59.88 µg/mL, which is lower compared to the nanoparticles synthesized without the ionic liquid (79.54 µg/mL), thus indicating a more pronounced cytotoxic effect of the ionic liquid-assisted nanoparticles in these cancer cells [134]. Nanoparticles of erbium oxide (Er_2_O_3_), iron oxide (Fe_2_O_3_), and their nanocomposite Er_2_O_3_/Fe_2_O_3_ have been synthesized successfully by the microwave method. The in vitro cytotoxicity of Er_2_O_3_ nanoparticles, Fe_2_O_3_ nanoparticles, and their nanocomposite was studied and compared using the MTT assay with the MDA-MB-231 human breast cancer cell line. The findings of the present study have revealed that the Fe_2_O_3_ nanoparticles, Er_2_O_3_ nanoparticles, and their nanocomposite Fe_2_O_3_/Er_2_O_3_ nanoparticles have shown significant cytotoxicity against the MDA-MB-231 human breast cancer cell line. However, it has also been found that the cytotoxicity of the nanoparticles is dose-dependent. After 24 h of incubation with different concentrations of the nanoparticles from 5 to 100 µg/mL, all the above formulations of the nanoparticles have significantly inhibited the viability of the cancer cell line compared with the control (100%). Additionally, Fe_2_O_3_-treated cells indicated that the maximum viability was only 20% at a concentration of 5 µg/mL. The Er_2_O_3_-treated cells showed that the viability was 17% at 50 µg/mL; the Fe_2_O_3_/Er_2_O_3_ composite showed that the viability was 21% at 75 µg/mL. Notably, although significant cytotoxicity was observed in the cancer cells MDA-MB-231, the nanoparticles showed a viability higher than 80% in normal cells MCF-10A, even at the highest concentration, thus showing selective anticancer activity and good biocompatibility. The results confirm the effectiveness of the synthesized nanoparticles in inhibiting the proliferation of breast cancer cells, possibly through apoptosis via internalization rather than membrane damage [135]. The anticancer effect of erbium is also reported by Wang et al., who investigated the tetraphenylporphyrin erbium acetylacetonate complex (Er(acac)TPP) as an anticancer agent by comparison with cisplatin in several cancer cell lines, including H1299, A549, and H460 lung cancer, NCI-H226 squamous cancer, HeLa cervical cancer, and HCT116 colon cancer, as well as 786-O renal carcinoma. The complex was also assessed in vivo in tumor mouse models. The results demonstrated that the Er(acac)TPP complex exhibited its greatest cytotoxic potency against the H1299 cancer cell line, with an IC_50_ value of 0.535 ± 0.019 µM. This value was considerably lower than that observed for cisplatin (21.59 ± 0.678 µM) for the same cell line, showing a higher efficacy of the erbium complex. For the 786-O renal carcinoma cell line, the IC_50_ values observed for the Er(acac)TPP complex (0.927 ± 0.066 µM) and for cisplatin (1.260 ± 0.131 µM) were similar, showing similar cytotoxicity of these compounds. The IC_50_ value for the H1299 cell line is 0.535 ± 0.019 µM, which is less than the IC_50_ value for normal cells, Beas-2b, which is 5.032 ± 0.122 µM. The IC_50_ results show that the compound is more toxic to the cancer cells than the normal cells. Er(acac)TPP has been observed to have a lower toxicity on the renal system compared to cisplatin, but more long-term observations are needed. In addition, the biological stability of the complex, which was tested for at least 48 h, and the use of the porphyrin ligand, known for its ability to preferentially accumulate in cancer tissues, contribute to the increased bioavailability [136]. Another erbium complex containing a Schiff base ligand (N^2^,N^3^-bis(anthracene-9-ylmethylene)pyridine-2,3-diamine) was synthesized in order to optimize erbium bioavailability and stability. Its anticancer effects were assessed in MCF-7 (breast cancer) and HeLa (cervical cancer) cells by comparison to normal Vero cells by using concentrations ranging from 5 to 100 µg/mL for a 24-h incubation period. A dose-dependent decrease in cell viability was reported. Although the range of the concentration has been shown graphically, the authors have discussed the concentration that corresponds to the IC_50_ value, which is around 25 µg/mL, and this concentration is considered to be biologically relevant. At this concentration, the percentage of cells that are viable is reduced to around 49% in MCF-7 cells and 42 to 51% in HeLa cells, thus showing a strong anticancer activity in vitro. In normal Vero cells, viability remained at approximately 65–75%, thus demonstrating moderate toxicity, yet lower than the one recorded in the tested tumor cell lines [137]. In a study conducted by Zaki et al., the antiproliferative activity of a cocaine erbium complex [Er(III)(Cn)Cl(H_2_O)_3_] · 2Cl was investigated in two cancer cell lines, MCF-7 and HepG-2, with cisplatin used as a positive control. The IC_50_ value calculated for the MCF-7 cell line was found to be lower when compared with the IC_50_ value of the control compound cisplatin (2.99 µM). Therefore, in HepG-2 cancer cells, the erbium complex showed lower antiproliferative activity than cisplatin as indicated by its significantly higher IC_50_ value [138]. Recent single-molecule investigations indicate that erbium exerts its anticancer potential primarily through strong, direct interactions with double-stranded DNA. In the case of physiological buffers, trivalent erbium (Er^3+^) has a high binding affinity to DNA, with association constants in the range of 106 to 107 M^−1^, and preferably targets the grooves of the DNA helix. However, at lower concentrations, this interaction has the ability to modulate the mechanical properties of the DNA, which leads to the promotion of local structural variations. Importantly, at higher concentrations, the interaction between DNA and the ion has the ability to cause significant compaction and condensation, leading to the eventual collapse of the DNA helix to a globular form. This type of cationic condensation is similar to the common polycationic condensing agents, which is the end product of charge neutralization and bridging caused by the negative phosphate backbone of the DNA molecule. Disruption of the DNA molecule has the capability to influence vital cellular processes, causing cytotoxicity among the cancer cells due to their high proliferative rate (Figure 2) [139].

## 10. Dysprosium

Dysprosium (Dy, Z = 66) has its own magnetic and optical properties because of the internal electrons. The most stable oxidation state for dysprosium is +3 (Table 2). This may explain the abundance of trivalent compounds such as oxides and fluorides in the chemical studies [140,141].

Dysprosium compounds, e.g., Dy_2_O_3_ and DyF_3_, are currently used in luminescent materials, lasers, and dosimeters in various industries (Figure 3) [142]. Dysprosium has also been assessed for its anticancer effects, as reported by Rotunjanu et al., who developed dysprosium-doped cobalt ferrite nanoparticles (CoFe_2−x_Dy_x_O_4_) containing different amounts of dysprosium and tested them in A375 (melanoma) and MCF-7 (breast) cancer cells. Cell viability dropped as the dose increased in both cancer cell lines. In A375 cells, hitting them with 1000 µg/mL brought viability down to about 43% for nanoparticles with x = 0.4. In comparison, undoped ferrite at that same dose left more cells alive—around 56 to 58%. Looking at breast cancer cells, the x = 0.4 formulation left 65% of the cells viable at 1000 µg/mL. However, with ferrite particles, cell toxicity started showing up at doses as low as 250 µg/mL. In addition, the formulation did not exhibit cytotoxic effects in HaCaT normal human keratinocytes, even when used in the highest concentration, thus revealing a high degree of selectivity against malignant cells. Furthermore, Western blot analysis demonstrated that cancer cell death occurred via a programmed mechanism, as evidenced by nuclear condensation and fragmentation, along with increased expression levels of Caspase-9 in the tumor cell lines, supporting the activation of the intrinsic apoptotic pathway [143]. Spinel-type ferrite nanoparticles with the composition Mn_0.5_Zn_0.5_Dy_x_Fe_2−x_O_4_ were investigated, in which dysprosium was introduced as a structural dopant into the ferrite lattice in order to evaluate the influence of the Dy content upon its biological properties. The in vitro anticancer activity in the HCT-116 human colorectal cancer cell line was assessed by means of MTT assay, with normal HEK-293 cells employed as a control. The results showed that the undoped Mn_0.5_Zn_0.5_Fe_2_O_4_ nanoparticles, as well as those containing low amounts of dysprosium (x ≤ 0.06), did not induce a significant decrease in cell viability, which indicates high biocompatibility. On the other hand, nanoparticles doped with higher amounts of dysprosium (x = 0.08 and 0.10) showed a marked inhibition of cell proliferation of HCT-116 cells, and the effect was found to be significant after 48 h of incubation and increased with increasing concentration of nanoparticles. At higher concentrations, these nanoparticles showed a marked reduction in the viability of tumor cells, whereas HEK-293 cells were found unaffected, showing selective cytotoxicity [144]. Dysprosium nanoparticles have shown toxicity to tumor cells and fewer side effects on healthy cells. There is no information available on the long-term toxicity of these nanoparticles. It has been observed that these nanoparticles are stable for 72 h in physiological pH, and in acidic environments, such as those of cancer cells, these nanoparticles degrade and release dysprosium [145]. When dysprosium oxide (Dy_2_O_3_) was structured into nanosheets via a green synthesis approach and evaluated against the A549 human lung adenocarcinoma cell line, a concentration-dependent reduction in tumor cell viability was reported, reaching approximately 22% inhibition at the highest tested concentration (500 µg/mL) and indicating a moderate cytotoxic effect that warrants further mechanistic and in vivo investigation [146].

The scientists made a compound with dysprosium, which is called Dy_2_(5-npic)_6_(H_2_O)_42_. They used this to see if it could stop cancer cells from growing. The dysprosium compound was tested on two types of cancer cells, prostate carcinoma cells and promyelocytic leukemia cells. After 24 h, the dysprosium compound killed about half of the prostate carcinoma cells when it was used at a concentration of 500 µg/mL. This shows that the compound can stop cancer cells from growing, and it does this in a way that depends on the dose. The scientists calculated that the IC_50_ value for prostate carcinoma cells was 427 µg/mL. However, for leukemia cells, the IC_50_ value was much higher at 819 µg/mL. This means that the dysprosium compound is not as effective against promyelocytic leukemia cells. These findings suggest that the dysprosium compound could be used to treat prostate cancer because it can kill cancer cells. More research is needed to fully understand how it works and to see if it is really effective [147].

Another study was done on a dysprosium compound, which is called Dy(phen-dion)_3_. This compound was found to produce oxygen species, which are molecules that can damage cells. It also interacts with the DNA in cells and causes genetic damage, which can lead to cell death. The anticancer activity of this compound was tested on colorectal adenocarcinoma cells. The results showed that the compound can stop these cancer cells from growing, and it does this in a way that depends on the dose. The IC_50_ value was found to be 10.16 µg/mL, which means that the compound is very effective at killing cancer cells. As the concentration of the compound was increased, more and more cancer cells were killed, with about half of the cells being killed at certain doses. This suggests that the dysprosium compound could be used to treat cancer by producing oxygen species that damage the DNA in cancer cells (Figure 2) [148].

In the study by Zou et al., the mononuclear Dy(III) complexes BrMQ-Dy and ClMQ-Dy were found to show potent anticancer activities in vitro on the human cancer cell lines HeLa, Hep-G2, BEL-7404, and MCF-7. Compared with BrMQ-Dy, ClMQ-Dy generally possesses higher cytotoxic activity, especially on the HeLa cell line, where IC_0_ = 1.01 µM. The IC_0_ values, which fall in the range of 1.01 to 29.56 µM, are in the micromolar concentration range, whereas the control dysprosium salt Dy(NO_3_)_3_ exhibited no observable cytotoxic activity, with IC_0_ > 150 µM. The complexes of Dy(III) have shown lower toxicity to normal HL-7702 cells compared to tumor cells. However, there is no information on the systemic toxicity of the complexes. There are no in vivo experiments in the article, and the pharmacokinetic profiles of the complexes are not studied. The experiments on HeLa cells have shown that the complexes were internalized efficiently. The ClMQ-Dy complex accumulated in a larger amount (7.59 ± 0.08 nmol Dy/10^6^ cells) compared to the BrMQ-Dy complex (5.81 ± 0.03 nmol Dy/10^6^ cells), which is related to its higher cytotoxic activity [149].

Dysprosium is studied in the biomedical field in complex form due to its low stability. Although its use in oncology is limited, some of its complexes have shown promising results in the treatment of cancer in vitro, such as those related to colorectal cancer cells, prostate cancer cells, HeLa cells, Hep-G2 cells, and BEL-7404 cells.

## 11. Europium

Located in the middle of the lanthanide series in the periodic table, with atomic number Z = 63 (Table 2), europium (Eu) is a rare-earth element with low toxicity and biological activity. It is mostly found in the trivalent form [150].

The earliest medical use of Eu was due to its fluorescence in cell imaging, quantitative detection, and monitoring of drug release behavior [151]. Eu-based materials have also been explored as theranostic agents, such as fluorescent nanohydrogels for drug delivery and heterometallic complexes for combining imaging and therapy [152,153].

More recent studies have shown that Eu exhibits anti-tumor, angiogenic, neuritogenic, antibacterial, and osteogenic properties, which have enhanced interest in its application in the biomedical field [151].

One study reported the development and utilization of Eu-doped CaF_2_ nanoparticles as intraosseous injection in vivo, which, when combined with X-ray irradiation, has shown satisfactory therapeutic effects in inhibiting primary tumor metastasis and controlling primary tumor size. In the same study, in vitro experiments show that CaF_2_:Eu NPs (200 µg/mL) produced selective toxicity in osteosarcoma cells (143B), including inhibition effects at an Eu dopant amount of 2.95 atomic weight percent, effects that were further enhanced under X-ray irradiation. Therefore, the results suggest that CaF_2_:Eu NPs may serve as a promising nanomedicine for adjuvant treatment of osteosarcoma following tumor resection [154].

Eu ion-doped superparamagnetic iron oxide incorporated into bovine serum albumin hybrid nano-complexes (Eu:SPIO@BSA nano-complexes) were developed to prevent metastatic osteosarcoma and to increase tumor accumulation through intravenous administration, and were afterward tested in vitro and in vivo. The in vivo studies demonstrated that Eu:SPIO@BSA nano-complexes inhibited distant lung metastasis, while the in vitro studies demonstrated a migration-inhibiting effect as well as the alterations in pseudopodia formation in K7M2 cells. According to the authors, the proposed mechanism of in vitro cytotoxicity, supported by previous studies, was most likely related to decreased matrix metalloproteinase-2 (MMP-2) protein expression, which is involved in extracellular matrix degradation before metastasis, while the detailed mechanisms underlying in vivo cytotoxicity require further clarification. These findings indicate that this nano-complex might be a useful therapeutic agent for improving osteosarcoma metastasis prevention. In terms of biosafety, Eu:SPIO@BSA nano-complexes revealed higher hemocompatibility and reduced liver toxicity compared to EuCl_3_·6H_2_O, findings that support their use as a safer therapeutic agent [155]. Chheda et al. synthesized europium oxide nanoparticles (Eu_2_O_3_ NPs) by using both sodium borohydrate and *Vitex trifolia* leaf extract as reducing agents. They later investigated their cytotoxic effects in A549 cancer cells. A dose-dependent decrease in cancer cell viability was noted, with an approximate IC_50_ value of 100 µg/mL, while normal cells showed lower toxicity. At high doses, cells exhibited marked morphological changes, severe stress, and a noticeable reduction in cell density. These effects are most likely attributed to ROS generation, a process facilitated by redox cycling between Eu^2+^ and Eu^3+^, which is enhanced under acidic tumor microenvironments and can lead to mitochondrial dysfunction, DNA damage, cell cycle arrest, apoptosis, or other effects reported in the literature [156].

Kotakadi et al. examined the cytotoxicity effects of different concentrations of Eu^3+^-doped hydroxyapatite nanocomposites (Han:Eu^3+^) on MCF7 and 4T1 breast cancer cell lines. The highest concentration of Han: Eu^3+^ NCs (200 µg/mL) decreased cell viability from 100% to 12.51%, and the IC_50_ was 54.43 µg/mL. The results of cell viability assays in MCF7 cells showed a concentration-dependent decrease in cell viability, thus indicating potential anticancer activity. Additionally, the results showed that while cell viability remained 100% in untreated cells, increasing concentrations of Han: Eu^3+^ NCs (6.25–200 µg/mL) caused a progressive decrease in 4T1 cell viability from 90.16% to 34.97%. A moderate cytotoxic potential was indicated by the estimated IC_50_ higher than 50 µg/mL [157].

Eu and terbium (Tb) complexes have been investigated for simultaneous imaging and anticancer applications; Bi et al. showed that Eu^3+^/Tb^3+^ loaded nano-micelles induced a significant cytotoxic effect in A375 melanoma cells that was further increased when loaded with additional anticancer drugs. However, Eu^3+^/Tb^3+^-loaded nano-micelles showed low toxicity toward normal human liver cells (L02). In the same study, drug-loaded Eu^3+^/Tb^3+^ nano-micelles effectively inhibited tumor growth in a murine melanoma model with minimal toxicity, and they enabled in vivo fluorescence tracking, showing tumor accumulation and retention. The micelles preferentially accumulated in tumor cells through interactions between the CD44 receptors on the tumor cells, supporting their potential for combined anticancer therapy and imaging [158]. Additionally, new Eu and Tb complexes and their Cu-containing binuclear analogs were synthesized, and their fluorescence behavior and cytotoxic effects were determined in HeLa cancer cells and A549 lung adenocarcinoma cells. These findings suggest that while their binuclear Cu-containing analogs exhibit increased anticancer activity, the Eu and Tb complexes showed only limited cytotoxicity but improved fluorescent parameters [159]

These findings indicate that europium may serve as a potential cancer treatment, either independently or in association with other anticancer therapies (Figure 2). However, its anticancer potential needs to be fully validated through further research.

## 12. Gadolinium

Gadolinium Gd is a soft, silvery-white metal that is a rare-earth element and a member of the lanthanide series, with atomic number Z = 64 (Table 2). It reacts with water and oxygen.

The trivalent gadolinium cation (Gd^3+^) has been administered to patients for over thirty years as a contrast agent in MRI, via the intravenous route, as it serves as the base of chelates. However, it may have harmful effects in patients with severe renal dysfunction, and it may also lead to tissue retention [160]. Furthermore, the theranostic effect of Gd in cancer therapy has been studied in previous years, as it possesses favorable imaging and therapeutic properties [161]. In addition, recent studies suggest that Gd compounds may also exert direct anticancer effects, and can also be used in the gadolinium-neutron capture therapy (Gd-NCT).

Zhang et al. investigated the effects of gadolinium oxide nanoparticles (Gd_2_O_3_ NPs) on autophagy processes together with their ability to strengthen the therapy with associated conventional drugs against human ovarian cancer cells. The results showed that Gd_2_O_3_ NPs are able to block the final phases of autophagy in a dose-dependent manner, finally resulting in autophagosome accumulation in HeLa cells. Moreover, Gd_2_O_3_ NPs enhanced the cytotoxicity of cisplatin in human ovarian cancer cells, suggesting their potential application in both diagnosis, due to their great potential for use as an MRI contrast agent, and the therapy of solid tumors [162]. In another study, bovine serum albumin (BSA)-coated gadolinium oxide nanoparticles (Gd_2_O_3_@BSA) were developed as a biocompatible carrier for curcumin (CUR), and their physicochemical characteristics and anticancer potential against nasal squamous cell carcinoma were evaluated. In vitro cytotoxicity assays revealed that Gd_2_O_3_@BSA-CUR demonstrated the greatest anticancer effect against RPMI 2650 and CNE-1 cancer cell lines, and the authors’ opinion was that the vehicle itself had a suppressive effect on cancer cell growth, suggesting that Gd may itself have potential anticancer effects. Furthermore, in vivo biosafety studies showed good biocompatibility of Gd_2_O_3_@BSA nanoparticles, with no mortality, no significant body weight changes, and no abnormal behavior observed at the tested doses. A histopathological evaluation also revealed a lack of major alterations, necrosis, or any sign of toxicity in the liver, kidney, spleen, or heart, thus confirming a favorable short-term safety profile. Therefore, Gd_2_O_3_@BSA may be regarded as a useful carrier for the delivery of anticancer drugs that is able to increase their therapeutic efficacy [163]. Man et al. obtained a Gd (III) compound (C4) with significant *T*_1_-weighted MRI signal and in vitro cytotoxic effects in cancer cells. They further enhanced its targeting, MRI performance, and tumor growth inhibition in vivo by developing an apoferritin-C4 (AFt-C4) nanoparticle system. C4 and AFt-C4 NPs inhibited tumor growth through apoptosis, ferroptosis, and ferroptosis-induced immune response [164]. Cao et al. designed and synthesized two Gd (III) complexes and investigated their antiproliferative effects as well as their activity on apoptosis and cell migration in A549 lung cancer cells. Their findings revealed that both compounds increased the production of intracellular ROS, caused dose-dependent apoptotic effects, and displayed stronger antiproliferative activity compared to cisplatin. Moreover, Gd1 and Gd2 inhibited tumor cell migration, and the cell cycle analysis revealed arrest in the G0/G1 phase. Western blot tests showed that both Gd complexes induced apoptosis by altering Bax and Bcl-2 protein expression [165].

Gd-NCT uses isotopically enriched gadolinium and thermal neutrons to specifically kill cancer cells [166]. Xie et al. explored the potential applications of Gd-DOTA-PSMA (Prostate-Specific Membrane Antigen) in Gd-NCT for prostate cancer treatment. The results demonstrated that 22Rv1 tumor-bearing mice administered Gd-NCT had significantly higher levels of the pro-apoptotic protein Bax, the tumor suppressor protein p53, and γ-H2AX. The study also discovered reduced expression of the proliferation marker PCNA and the anti-apoptotic protein Bcl-2, suggesting that the treated group was promoting apoptosis and preventing tumor growth. The tumor tissues showed signs of cellular death and fragmentation, while no histological abnormalities were detected in the major organs, including the heart, liver, spleen, lungs, and kidneys, indicating good in vivo biocompatibility. Elevated γ-H2AX levels indicated increased DNA damage in the group treated with Gd-NCT. Gd-DOTA-PSMA can act as a novel theranostic bio-gadolinium-base agent for the treatment of prostate cancer and has shown considerable promise for targeted neutron capture therapy [166].

In another study, researchers developed a stem cell-nanoparticle system (SNS) by magnetizing umbilical cord mesenchymal stem cells (UMSCs) with gadodiamide-concealed magnetic nanoparticles (Gd-FPFNP) to actively target glioblastoma multiforme (GBM) and overcome the limitations of gadolinium-based agents for neutron capture therapy (Gd-NCT) in this type of cancer. The SNS successfully addressed the limitations of conventional Gd agents, resulting in significant tumor inhibition and extended survival after a single NCT treatment with an ultra-low Gd dose. Systemic and local toxicity studies conducted in C57BL/6 mice during 14 days showed a suitable safety profile; the animals exhibited stable body weight, normal clinical signs, lack of neuronal degeneration, and the absence of histological abnormalities in major organs, such as the heart, liver, spleen, lungs, kidneys, and brain. These results highlight SNS as a promising strategy for Gd-NCT in GBM, with potential applicability to other tumor types [167]. Tokumitsu et al. investigated gadolinium neutron-capture therapy (Gd-NCT) for cancer using chitosan nanoparticles as a novel gadolinium delivery system. These nanoparticles contained 1200 µg of natural gadolinium and were injected intratumorally twice in mice bearing subcutaneous B16F10 melanoma. After the second administration, thermal neutron irradiation was performed on the tumor site. The results showed that tumor growth was significantly inhibited after irradiation in the nanoparticle-administered mice, compared to mice administered with gadopentetate solution, regardless of the smaller Gd dose compared to that used in previous research and the radioresistance of melanoma. According to this study, gadolinium-loaded nanoparticles could be utilized as Gd-NCT [168].

In conclusion, Gd-based compounds exhibit both direct anticancer effects and theranostic potential (Figure 2), with promising applications in Gd-NCT, based on the studies mentioned above. However, more investigation is needed to confirm these findings.

## 13. Holmium

Holmium (Ho) is a rare-earth element with atomic number 67 (Table 2), discovered first in the 1870s. The radioactive isotope holmium-166 can be produced by neutrons activating the naturally occurring isotope holmium-165, which is stable and makes up 100% of holmium in nature [169]. Holmium can be observed with CT and MRI since it has paramagnetic characteristics and a high attenuation coefficient [170].

In a systematic review and meta-analysis, Sólymos et al. evaluated transarterial radioembolization with Holmium-166 microspheres (Ho-166-TARE) as a therapeutic approach that delivers radiation directly to liver tumors, while also enabling precise imaging and personalized dosimetry (Figure 3). Their findings indicate that patients with incurable primary or metastatic liver cancers may benefit from Ho-166-TARE as a targeted oncologic therapy for liver malignancies. Ho-166-TARE also showed favorable survival outcomes with negligible side effects [171].

Munaweera et al. incorporated holmium-165 together with platinum-based chemotherapeutics into garnet magnetic nanoparticles for magnetic tumor targeting (referred to as HoIG for holmium-incorporated garnet nanoparticles) and evaluated the anticancer potential of these nanoparticles both in vitro and in vivo. The findings indicated that holmium-containing garnet nanoparticles exhibited minimal cytotoxicity towards NSCLC A549 cells. On the other hand, combining HoIG with cisplatin greatly increased toxicity against the same cell line, more than free cisplatin alone. In animal studies, researchers found that applying an external magnetic field led to more holmium being directed to the tumor site. In animal studies, researchers found that applying an external magnetic field led to more holmium being directed to the tumor site. This was clear from higher tumor-to-liver ratios, which showed that the nanoparticles accumulated in the tumor. As a result, tumor growth was significantly reduced compared to control [172].

In another study, holmium oxide (Ho_2_O_3_) nanoparticles were investigated in terms of cytotoxic effects in 3T3 and MCF-7 cell lines. The results showed that the Ho_2_O_3_ nanoparticles were more effective as antiproliferative agents in 3T3 cells than in MCF-7 cells. The authors reported that the anticancer mechanism of rare earth lanthanides is mostly attributed to oxidative stress induced by ROS, calcium transport disruption, direct interactions with cellular organelles and DNA, endoplasmic reticulum stress, and the activation of MAPK pathways (Figure 2). Moreover, an *in ovo* antiangiogenic assay in the same study indicated that the nanoparticles could inhibit the development of new blood vessels, suggesting potential applications in restricting tumor angiogenesis. These biological effects were dose-dependent [173].

Zhang et al. developed a multifunctional Ho(III)-doped mesoporous polydopamine nanosystem, coated with 4T1 cell membranes and loaded with mitoxantrone, for MRI-guided chemo-photothermal therapy. This system showed better selective uptake in vitro in 4T1 breast cancer cells, where it caused dose-dependent cytotoxicity, apoptosis, and necrosis, especially after NIR laser irradiation. In Hepa1–6 liver cancer cells, the cytotoxic effects were less pronounced. In contrast, normal HUVEC cells lacked cytotoxic effects and remained viable, further supporting the high biocompatibility of the nanosystem. In vivo studies in 4T1 tumor-bearing mice revealed significant tumor growth inhibition, accompanied by the reduced expression of Ki67 and CD31 and extensive apoptosis and necrosis. Moreover, the nanosystem showed negligible hepatic, renal, and systemic toxicity, further highlighting its favorable safety profile [174].

Bult et al. synthesized holmium-166 acetylacetonate microspheres and evaluated their use in an orthotopic mouse model as a local treatment for renal cancer. The application of the microspheres in mice resulted in almost complete control of the tumor growth, while the untreated group showed rapid tumor progression. Histological analysis revealed focal necrosis, infiltrations of inflammatory cells, and extensive tumor cell death 24 h after the treatment, effects that were mainly attributed to the local delivery of beta radiation from holmium-166, resulting in effective tumor ablation via high absorbed radiation doses. Also, no significant radiation damage was observed outside of the treated area, with the surrounding healthy renal tissue remaining histologically normal, which suggests a favorable safety profile for this Ho-based radiotherapeutic strategy [175].

Further evidence comes from Zambanini et al., where the authors developed an injectable system for the treatment of bone cancer that used bioactive glass particles with holmium in a hydrogel (Poloxamer 407). This system promoted the proliferation of pre-osteoblastic MC3T3-E1 cells while decreasing the viability of MG-63 osteosarcoma cells, with the 5 wt.% Ho_2_O_3_ formulation exhibiting optimal results. The anticancer effect was primarily associated with the ions released from the glass matrix, particularly calcium ions, whereas Ho^3+^ may also facilitate osteoblastic proliferation and bone regeneration. The system demonstrated excellent biocompatibility and appropriate characteristics for injectable delivery, indicating its potential as a safe method for bone cancer treatment [176].

## 14. Lanthanum

Lanthanum (^139^La), which is a trivalent rare-earth metal (Table 2), has, in the last few years, somehow become a kind of strange biological actor in cancer research, both as a simple ion and in more complicated nanomaterial forms that scientists keep designing and redesigning.

Some studies have shown that lanthanum and its nano versions do not just damage DNA directly; they also create reactive oxygen species and disturb the signaling routes that cancer cells depend on, and they even seem to change how the tumor microenvironment reacts (Figure 5) [177]. At the molecular level, lanthanum salts can slow down cell growth through very specific pathways, though sometimes the data feel inconsistent. For instance, lanthanum citrate was reported to stop proliferation and cause apoptosis in hepatocellular carcinoma SMMC-7721 cells by blocking the Hedgehog pathway. In those experiments, Gli1 and Sonic hedgehog went down, both in mRNA and protein forms, while Cyclin D1 and Bcl-2 also dropped, but p21 and cleaved Caspase-3 went up [178]. Therefore, it is not just a toxic ion; it behaves like a signaling manipulator—one that can silence developmental pathways that tumors hijack. Beyond its basic toxicity, lanthanum salts seem to make chemotherapy work better. In ovarian cancer cells, lanthanum chloride rendered cisplatin more effective as an anticancer agent, even when resistant SKOV3 cells were used. The treatment blocked the PI3K/Akt pathway and reduced RAD51 expression, which interfered with DNA repair by homologous recombination. It also pushed cells toward apoptosis, with higher Bax and cleaved caspase-3 and lower Bcl-2 [179]. The drug damages the DNA in cancer cells, making it harder for the cells to repair the damage (Figure 2). This effect is especially important for metal-based cancer treatments, where getting around resistance to platinum drugs is still a big problem [177]. La_2_O_3_ nanoparticles inhibited the proliferation of osteosarcoma MG63 cells while enhancing the viability of normal Vero cells [180]. This dual biological behavior suggests that lanthanum-induced oxidative stress mostly affects tumor cells that exhibit high levels of basal ROS and a low ability to buffer redox reactions. This type of toxicity, which depends on the environment, makes lanthanum nanomaterials more appealing for clinical applications due to their potential to leave other targets unharmed. Structural engineering of lanthanum nanoplatforms has facilitated tumor microenvironment-responsive therapy. A dendritic mesoporous lanthanum-based nanoplatform engineered for glioblastoma treatment exhibited selective dissociation in mildly acidic tumor microenvironment conditions, facilitating the localized release of lanthanum ions. When exposed to near-infrared light, this platform showed good photothermal conversion and increased ROS production, which led to synergistic oxidative and photothermal tumor suppression both in vitro and in vivo. The release of ions caused by the acidity of tumors and the activation of photothermal therapy shows how lanthanum-based systems can be both responsive to the environment and effective at treating multiple types of cancer [181]. Lanthanum-based MOFs (Metal–Organic Frameworks) have many flexible coordination environments. This lets you control how much of the drug is loaded and released [182]. Lanthanum-doped ZnO quantum dots were more fluorescent and toxic to cells when treated with doxorubicin than undoped ZnO systems. The La-ZnO@DOX formulation worked better against tumors in both lab tests, which suggests that adding lanthanum changes both the photophysical and biological properties. Lanthanum can improve both optical and therapeutic properties, showing how important it is in theranostic design [183]. Adding tumor-specific ligands to the drug allows it to selectively build up in the tumor microenvironment.

The broader theranostic potential of lanthanide nanoparticles, including lanthanum-based systems, has been comprehensively discussed in recent analyses of molecularly targeted nanomaterials. Adding tumor-specific ligands allows for selective accumulation in the tumor microenvironment, and the intrinsic magnetic, optical, and radiological properties support multimodal imaging and image-guided therapy [184].

In terms of toxicological profile, La is mostly investigated as orally administered lanthanum carbonate, which is used as a phosphate binder in the treatment of chronic kidney disease with hyperphosphatemia. Therefore, the most reliable data does not come from nuclear medicine but from nephrology. Generally, lanthanum carbonate showed low systemic bioavailability combined with high long-term clinical safety. It mainly exhibits local gastrointestinal activity, although certain tissue accumulation may occur under particular conditions, such as uremia. In a phase I human study, lanthanum carbonate revealed a very low absolute bioavailability and insignificant renal clearance, which indicates minimal systemic absorption and an elimination through non-renal mechanisms, presumably hepatobiliary [185]. In agreement with this study, another preclinical work reported a biliary excretion of La in rats [186]. In hemodialysis patients, La carbonate was generally well tolerated, exhibiting only local gastrointestinal side effects, without liver, bone, or central nervous system toxicity [187]. Earlier data also revealed sustained tolerability following several years of exposure [188]. Also, under current treatment conditions, the standard phosphate-binder therapy did not produce any adverse cognitive effect, therefore supporting the hypothesis that La carbonate is not able to effectively penetrate the blood–brain barrier [189]. Nonetheless, La cannot be considered biologically inactive. Experimental studies revealed that, under uremic conditions in chronic renal failure models, La exhibits tissue accumulation following oral administration, particularly in the liver, bone, and kidney, but also in other organs [190]. Additional in vivo experiments revealed a progressive liver [191] and bone surface deposition [192]. Nanoparticles containing La titanate have shown preliminary in vivo compatibility in short-term studies conducted in mice, but data remained limited and much less clinically relevant compared to La carbonate [193]. However, collectively, data show that La biocompatibility depends on its route of administration; orally, it is relatively safe due to its low bioavailability, but its long-term safety is closely related to its formulation, the patient’s renal integrity, and the potential of slow tissue deposition as a result of chronic exposure.

## 15. Lutetium

Lutetium (^177^Lu) is currently regarded as one of the most important radionuclides used in modern oncology, primarily because of its favorable physical characteristics (Table 2), stable radiochemistry, and its proven clinical benefits in the treatment of advanced malignancies. With a half-life of approximately 6.7 days, this medium-energy β^−^ emitter is able to deliver sustained radiation to the tumor tissue while maintaining a relatively short penetration range in the soft tissue (approximately 0.5–2 mm). This limited penetration allows efficient destruction of tumor cells while minimizing damage to surrounding healthy structures.

In addition, the low-energy γ emissions of ^177^Lu allow post-therapy imaging and dosimetric evaluation, thereby supporting the integration of treatment and monitoring within a single theranostic strategy. Owing to these characteristics, ^177^Lu has become a central component of radioligand therapy (RLT), particularly in malignancies that overexpress molecular targets capable of selective ligand binding [194].

The clinical value of ^177^Lu was clearly reported in neuroendocrine tumors (NETs), where it was used in the peptide receptor radionuclide therapy (PRRT) that targeted somatostatin receptors (SSTRs) (Figure 4). The phase III NETTER-1 trial and its subsequent dosimetric analyses reported that the repeated administration of [^177^Lu]Lu-DOTATATE is feasible and safe within the established organ dose limits. Patients who received four treatment cycles of 7.4 GBq showed cumulative absorbed doses to the kidneys and bone marrow that remained within accepted safety limits, indicating that fixed-activity protocols are generally well tolerated in advanced midgut NETs. The tumor-absorbed dose also varied significantly between patients. These differences may be caused by variations in the SSTR expression between tumors and emphasize the potential benefit of personalized dosimetry in improving treatment outcomes [195].

Fibroblast activation protein (FAP) has recently attracted considerable interest as a pan-tumor target because it is widely expressed in cancer-associated fibroblasts across many solid tumors. In a phase I/II study evaluating [^177^Lu]Lu-satoreotide tetraxetan, a somatostatin receptor antagonist, patients with advanced SSTR-positive NETs received several treatment cycles and showed favorable tumor uptake, with disease control rates exceeding 90%. The therapy was well tolerated, with manageable hematologic toxicity and no signs of clinically significant kidney toxicity. These results suggest that ^177^Lu can be incorporated into various molecular targeting strategies, broadening its therapeutic potential in NETs [196].

An evaluation of nearly 4000 safety reports from the WHO global database (VigiBase) found that hematologic toxicities, particularly anemia and thrombocytopenia, are the most frequently reported adverse events. Renal impairment and secondary myeloid neoplasms appear to be much less common, although they still require careful monitoring. Overall, the agreement between findings from clinical trials and post-marketing surveillance adds further confidence in the reliability of ^177^Lu-based therapy in routine clinical practice [197].

^177^Lu has demonstrated significant clinical efficacy in metastatic castration-resistant prostate cancer, where the therapeutic focus is on prostate-specific membrane antigen (PSMA), a transmembrane protein that is highly expressed in advanced prostate tumors. In the phase III VISION trial, patients who had previously received androgen receptor pathway inhibitors and taxane-based chemotherapy were treated with ^177^Lu-PSMA-617 in addition to standard care. These patients achieved a median overall survival of 15.3 months, compared with 11.3 months in the control group, corresponding to a hazard ratio for death of 0.62. Radiographic progression-free survival was also significantly longer, indicating that ^177^Lu-PSMA-617 can function as an effective systemic therapy capable of extending survival in heavily pretreated mCRPC, rather than serving only as a palliative radiologic intervention. At the cellular level, PSMA-targeted radioligands bind to PSMA on the surface of tumor cells and are then internalized through receptor-mediated processes. β radiation is delivered directly inside the tumor cells, where it generates DNA double-strand breaks that ultimately trigger apoptotic cell death. The emitted β particles also produce a crossfire effect, allowing radiation to reach nearby tumor cells even when PSMA expression is not uniform. The therapy can partially compensate for intratumoral heterogeneity and improve treatment coverage within metastatic lesions [198,199].

With the approval of lutetium ^177^Lu vipivotide tetraxetan, PSMA-targeted radioligand therapy became available for clinical use. The suggested dosing schedule is 7.4 GBq every six weeks for up to six cycles, with changes to the dose based on blood and kidney tests [199]. Most adverse events are hematologic, such as anemia, thrombocytopenia, and leukopenia. They can usually be managed with supportive care and changes to treatment. Xerostomia, caused by physiological PSMA expression in the salivary glands, is a known but usually mild form of toxicity.

Researchers have also started looking into using ^177^Lu-PSMA therapy in the early stages of the disease, and the first results are promising. Specifically, neoadjuvant therapy with ^177^Lu-PSMA-617 has been investigated in patients with high-risk localized prostate cancer prior to radical prostatectomy. Early clinical studies have shown that preoperative radioligand therapy lowers prostate-specific antigen (PSA) levels significantly and causes some pathological responses. Nevertheless, total pathological remission following a solitary treatment cycle seems to be infrequent. These results suggest that we may need to improve the way we dose, the number of treatment cycles, and possible combination approaches in order to better control tumors in localized disease [200].

Fibroblast activation protein (FAP) has gained considerable attention as a potential pan-tumor target because it is expressed in cancer-associated fibroblasts in many solid malignancies. In a phase II study evaluating ^177^Lu-LNC1004 in heavily pretreated patients with end-stage metastatic cancers, radioligand therapy achieved disease control in approximately 46% of participants. The treatment was applied with 3.33 GBq per cycle and was generally associated with moderate hematologic toxicity. No severe renal or hepatic adverse effects were reported. The mean tumor absorbed dose of 4.69 Gy/GBq indicates the effective tumor targeting, with a negligible effect in healthy organs. Overall, these results indicate that ^177^Lu-based radioligand therapy could extend beyond organ-specific indications and may serve as a flexible platform adaptable to a variety of molecular targets [201].

The immunological effects of the β-emitting radioligands have attracted increased interest. The preclinical investigations on a combination of ^177^Lu-DOTA-2P(FAPI)_2_ and PD-L1 checkpoint inhibition revealed an enhanced CD8^+^ T-cell infiltration, increased immunogenic cell death, and persistent tumor regression in murine models [202]. These results indicate that radiation-induced antigen release and immune activation may convert immunologically “cold” tumors into responsive microenvironments, thereby providing a rationale for immunoradiotherapy combinations. The combination of targeted β radiation and immune checkpoint blockade is emerging as a promising strategy in translational oncology.

The use of nanotechnology has further enriched the therapeutic potential of ^177^Lu. In HER2-positive breast cancer models, ^177^Lu-labeled trastuzumab-functionalized mesoporous Carbon@Silica nanostructures showed a high radiochemical yield of about 93% and selective uptake in HER2-overexpressing SK-BR-3 cells. After 24 h, cellular uptake reached nearly 44%, whereas it was only around 21% in HER2-negative MDA-MB-231 cells. The IC_50_ values were in the low micromolar range in receptor-positive cell lines, supporting receptor-mediated specificity. These results show that it is possible to combine antibody targeting with ^177^Lu radiolabeling in nanoparticle-based systems to improve how long the particles stay inside cells and how well they target tumors [203].

From a toxicological perspective, Lu biological behavior is highly determined by its chemical specie, such as free ions, chelated complexes, or nanostructured products. Subsequently, the most reliable information in terms of long-term toxicity derives from the clinically approved radiopharmaceuticals, particularly containing ^177^Lu, instead of the pure element or its inorganic salts. For example, within the peptide receptor radionuclide therapy (PRRT), ^177^Lu-DOTATATE was investigated in large cohorts and revealed an overall high safety. However, there are still certain side effects that, although rare, may pose clinical challenges, such as the myelodysplastic syndrome and acute leukemia [204]. Renal toxicity, which is generally a dose-limiting factor within radiotherapy, is less severe for 177Lu compared to other beta emitters such as 90Y, particularly in the presence of amino acids that are administered simultaneously [205]. Similar data were also published for the ^177^Lu-PSMA-617 therapy against prostate cancer that showed mainly hematologic toxicity, with limited evidence in terms of systemic effects attributable to Lu [206]. Therefore, one may state that, clinically, toxicity associated with Lu is rather determined by the radiation dosage and biodistribution than its intrinsic toxicity as a heavy metal.

Its metabolic clearance does not follow the conventional hepatic pathway but the one specific to the carrier molecule. When low molecular weight radioligands such as DOTATATE or PSMA inhibitors are used, Lu elimination takes place through renal excretion. Dose-related studies revealed Lu causing significant kidney, salivary glands, and tumor tissues uptake, with the clearance rate dependent on the ligand affinity and molecular size [207,208]. Conversely, agents targeting bones, such as ^177^Lu-EDTMP, accumulate preferentially in the skeletal tissues from where the systemic clearance follows pathways specific to the targeted tissues, with mild, dose-dependent hematological toxicity [209].

When Lu is used as oxide or hybrid nanoparticles, its biological fate is strongly determined by the particle size, surface functionalization, and colloidal stability. For example, PEG-ylated nanoparticles containing Lu showed favorable pharmacokinetic behavior that included reduced nonspecific protein binding and systemic clearance within weeks. Thus, one may state that the suitable surface tuning ensures high biocompatibility [210]. Other studies regarding ligand-functionalized ^177^Lu-labeled nanosystems reported only low acute toxicity and high in vitro stability, thus indicating such nanoparticles as potential theranostic platforms [211,212].

Overall, Lu exhibits a relatively high biocompatibility, with the highest safety reported for chelated radiopharmaceuticals and surface-engineered nanoparticles. Its long-term toxicity is limited but present, represented by bone marrow and renal toxicity following its systemic administration. The side effects are mainly caused by radiation exposure and pharmacokinetic profiles instead of its intrinsic toxicity as a heavy metal ion. Therefore, its safety profile can be further optimized by an improved chelation chemistry, increased target specificity, and pharmacokinetic parameters.

## 16. Neodymium

Neodymium (Nd^3+^), a lanthanide chemical element, has several interesting properties that make it useful for biological and photoluminescent applications. It is one of the most often employed elements in the formation of crystalline complexes (Table 2) [213].

Nd^3+^ ions have been used to diagnose diseases, for cancer therapy, and to fight bacterial infections [214]. Its optical properties also make it feasible to make fluorescent markers that may be used to watch cells and proteins in cell biology research [215]. Several Nd^3+^ complexes with potential anticancer properties have been developed, with some of them currently undergoing clinical trials, while others are already used as diagnostic and therapeutical agents [216].

Unlike platinum-based drugs that function mainly through DNA crosslinking, neodymium-based compounds exhibit a wider range of biological interactions, including DNA binding, induction of oxidative stress, mitochondrial dysfunction, and photothermal or imaging-assisted therapy optimization. The study of neodymium oxide nanostructures, Nd^3+^ coordination complexes, and Nd-doped magnetic nanoparticles revealed that this element can reduce cancer cell viability via mechanisms that involve the generation of reactive oxygen species (ROS), the regulation of apoptotic genes, and the structural alteration of nucleic acids (Figure 2) [215,217].

Neodymium oxide (Nd_2_O_3_) nanostructures have been examined for their potential cytotoxic effects in human cancer cell models. In studies performed on HepG2 (hepatocellular carcinoma) and A549 (lung adenocarcinoma) cell lines, curved Nd_2_O_3_ nanostructures, with diameters of around 140 nm and lengths reaching up to 700 nm, showed a clear dose-dependent toxic effect. Their impact on cell viability was evaluated using MTT and NRU assays. Treatment with Nd_2_O_3_ nanoparticles led to a noticeable decrease in cell viability and produced several molecular changes typically associated with apoptosis. Quantitative RT-PCR analysis revealed an increased expression of pro-apoptotic genes, including p53, Bax, and caspase-3, while the expression of the anti-apoptotic gene Bcl-2 was reduced [217]. At the same time, the cells showed higher levels of intracellular reactive oxygen species (ROS), along with noticeable changes in cell-cycle distribution.

Nd^3+^ coordination complexes have recently been investigated as potential metallodrugs, largely due to their selective behavior and their ability to exhibit several biological activities. These complexes can interact with DNA molecules with relatively high equilibrium association constants. They may promote the condensation of the DNA helix and trigger apoptosis in breast cancer cells. Some Nd^3+^ complexes containing coumarin have been shown to be highly cytotoxic, with IC_50_ values within the micromolar range. In some cases, their activity was even higher than that of similar complexes with La^3+^ or Ce^3+^. These Nd^3+^ complexes can attach to DNA by either inserting themselves between base pairs or electrostatically binding to the phosphate backbone. This could block important processes like DNA replication and transcription [215].

Magnetic nanomaterials containing neodymium have recently been investigated for their potential use in cancer research. In one example, Nd-doped cobalt ferrite nanoparticles (CoFe2-zNdzO4) synthesized through combustion formed a stable single-phase spinel structure that induced quantifiable cytotoxic effects against several cancer cell lines, including A375 melanoma, MCF-7 breast adenocarcinoma, and PANC-1 pancreatic carcinoma. In vitro tests indicated a dose-dependent cell viability decrease at concentrations ranging between 50 and 1000 µg/mL. Visible changes in cell morphology, including the disruption of the cytoskeleton and alterations of the nuclear structure specific for apoptotic processes, were reported. Interestingly, cancer cells seemed to be more responsive to these nanoparticles than normal keratinocytes (HaCaT), which suggests a certain degree of selectivity. The biological effects are believed to be linked to heightened ROS production, caspase-3 activation, alterations in mitochondrial membrane permeability, and DNA fragmentation, mechanisms typically associated with apoptosis induced by metal-based nanoparticles [218].

Neodymium is increasingly being explored in image-guided cancer research because it can be incorporated into multifunctional diagnostic nanoparticles. Experimental data showed that such nanoparticles do not significantly reduce the viability of human breast cancer cells, thus suggesting high biocompatibility and supporting their potential use in diagnostic applications. Nd^3+^-doped nanoparticles allow the simultaneous use of different imaging techniques, such as fluorescence imaging, photoacoustic imaging, and MRI [218]. The ^4^I_9_/_2_ → ^4^F_5_/_2_ transition in Nd^3+^ ions enables non-radiative relaxation pathways that transform optical energy into thermal energy, thereby facilitating photothermal tumor ablation. Nd^3+^-based nanoparticles lower water absorption and tissue overheating compared to Yb^3+^-based systems that need 980 nm excitation. This makes biological applications safer [219]. Nd^3+^-doped systems can integrate photothermal conversion with imaging and possibly radiosensitization, situating neodymium within multifunctional oncological nanomedicine platforms. Evidence indicates that neodymium exhibits anticancer properties through multiple coordinated mechanisms, including ROS-mediated apoptosis, mitochondrial dysfunction, caspase activation, DNA structural disruption, and, in engineered nanoplatforms, photothermal or imaging-assisted enhancement. It has been shown that Nd^3+^ coordination complexes exhibit inherent cytotoxic effects, and Nd_2_O_3_ nanostructures can cause oxidative and genotoxic stress in liver and lung cancer cells. Additionally, Nd-doped magnetic nanoparticles exerted dose-dependent antiproliferative effects in melanoma, breast, and pancreatic cancer cells [217,218].

Although they revealed anticancer activity, compounds containing neodymium represent significant challenges in terms of long-term toxicity and biocompatibility. Several studies reported that prolonged exposure to neodymium is able to trigger oxidative stress, inflammatory reactions, and cellular damage in both cancer and normal cells [213,220]. An increased intracellular ROS production appears to be the main mechanism underlying such effects, finally leading to lipid peroxidation, protein modification, and DNA damage (Figure 5) [220]. In addition, neodymium oxide nanoparticles have been associated with mitochondrial dysfunction, altered membrane integrity, and permanent alteration of the cellular homeostasis [213]. At the molecular level, neodymium is able to modulate the expression of genes involved in apoptosis and stress-response pathways, which may, in turn, contribute to both its anticancer activity and its cytotoxic effects on healthy tissues [220]. Furthermore, challenges related to its bioaccumulation and long-term retention due to limited clearance remain insufficiently understood [220] and require further studies.

## 17. Praseodymium

Praseodymium, a rare-earth element with Z = 59, is of particular interest for biomedical research because it displays electron shells with unfilled electrons and multiple oxidative states (Table 2), other than the +3 state, characteristic of lanthanide metals; it can also exist in the +4 and +5 states [221,222].

Lanthanide complexes, where the metal is in the +3 oxidation state, have shown anticancer activity [223], albeit praseodymium (IV) complexes, representing a new class of compounds, and the data on their antitumor activity is scarce [224]. For instance, Aly et al. studied the effects of the polymeric praseodymium (IV) and N-acetyl anthranilic acid complex against A375, HepG-2, A549, MCF-7, MDA-MB-231, PC-3, and T-24 cancer cell lines, respectively, but no promising cytotoxic activity was observed [224]. Similarly, praseodymium’s higher oxidation state (V) was considered unreachable for a long period of time, given its challenging synthesis [222]. The recent formation of praseodymium (V) complexes indicates a progression in the chemistry field [225]. Alternatively, there are many studies that describe the activity of praseodymium (III) compounds. Namely, Pradeep et al. synthesized a binuclear, ten-coordinated, hydrated praseodymium (III) EDTA phenyl hydrazine complex, in which the praseodymium +3 oxidation state was confirmed by magnetic measurements. Along with antiviral and antioxidant activity, the complex showed a 96.7% cytotoxicity against neuroblastoma cells at 50 µg/mL concentration [226]. Furthermore, Andiappan et al. described a praseodymium (III)-based Schiff base ligand complex and its activity against MCF-7, HeLa, and Vero cell lines. At lower concentrations, the viability of healthy Vero cells remained high, although it decreased when higher concentrations were tested. Nevertheless, the authors described the compound as more biocompatible with the Vero line compared to the erbium-based Schiff base complex. In cancerous cells, cell viability started to decline to under 50% when the 25 µg/mL concentration of the tested compound was applied, with the strongest cytotoxic effect noticed in breast cancer cells. A rise in ROS generation, subsequent apoptosis, and DNA fragmentation were identified as the underlying mechanisms of the antitumor activity (Figure 5). The praseodymium (III)-based Schiff base complex has been described as cytocompatible, stable, and able to bind with biomolecules (DNA), thus representing a potential anticancer drug [137]. Similarly, with the help of a tridentate Schiff base ligand, Abdel-Fatah et al. synthesized a praseodymium (III) complex, [PrBCl(H_2_O)_2_]·nH_2_O. The complex was characterized using infrared spectroscopy, 1H NMR, mass spectrometry, elemental analysis, magnetic susceptibility, conductivity analysis, PXRD, and thermal analysis. Moreover, its anticancer potential was tested against the HepG-2 cell line. The nonelectrolytic, paramagnetic formulation exhibited a dose-dependent cytotoxic effect, with the highest tested concentration (500 µg/mL) reducing cell viability to below 20%. In addition, the experiments showed that the complex exerted stronger anticancer activity compared to the free praseodymium. From the mechanistic point of view, the authors focused on praseodymium’s antimicrobial activity, leaving its cytotoxic effects for future research [227]. Bellot et al. described another mechanism presumably responsible for the anticancer activity of the praseodymium complexes. Such a coordinate complex of praseodymium with mercaptopyridine oxide has been shown to target lysosomal activity and affect mitochondrial metabolism in HeLa, MCF-7, MDA-MB-231, HCT116 WT, HCT116 *Bax/Bak DKO* and HCT116 *p53 KO* cancerous cells, while significantly reducing the viability of the respective cells. Moreover, it was proposed that the complex acts as a Zn^2+^ ionophore, influencing the zinc-mediated toxicity: it promotes Zn^2+^ influx into the cell, thus disrupting cellular survival processes [226].

Along with praseodymium complexes, many researchers focused on the synthesis and evaluation of praseodymium-based nanomaterials. When tested in A375 (melanoma), MCF-7 (breast cancer), and HT-29 (colorectal carcinoma) cells, praseodymium (III)-doped cobalt ferrite nanoparticles, containing different concentrations of praseodymium (CoFe_2−y_Pr_y_O_4_, y = 0–0.2), showed promising antiproliferative effects. Namely, the cytotoxic activity was dose-dependent, and it increased with the amount of praseodymium in the respective formulation. The authors suggested that the praseodymium substitution level, as well as surface properties and morphological characteristics, could all contribute to antiproliferative activity (Figure 2); however, these aspects require future investigations [228]. Another study has shown that praseodymium (III) substituted Ni-Co nano-spinel ferrites (Co_0.5_Ni_0.5_Pr_x_Fe_2−x_O_4_ (0.0 ≤ x ≤ 0.10) reduced the viability of HCT-116 colorectal cancer cells, although the IC_50_ values were not linearly correlated to the amount of praseodymium in the formulation. The treated cells showed signs of chromatic fragmentation and nuclear disintegration, suggesting apoptosis. However, the compound also decreased the viability of healthy human embryonic kidney cells HEK-293, although in a reduced manner compared to cancerous cells, thus showing a certain degree of selectivity as an anticancer agent. Nevertheless, the authors described the formulation as biocompatible [229]. In terms of toxicity, several studies emphasize the role of dose and exposure. For example, in *S. cerevisiae,* toxic effects were expressed through growth inhibition, which was time and dose dependent [230]. Similarly, the impact of dose on toxicokinetics was observed in Sprague–Dawley rats: at initial exposure, the half-live of the lanthanide increased, and fecal elimination decreased with the rise of dosage. At later phases, this correlation was no longer observed. However, it was mentioned that praseodymium accumulates in several organs, mainly in the liver, followed by spleen, kidneys, and lungs [231].

Moreover, Azhagar et al. synthesized two praseodymium oxide nanoformulations, which were then tested in MCF-7 breast cancer cells. Due to its +3 and +4 oxidation states as well as the lack of one oxygen atom, praseodymium oxide has the ability to generate reactive oxygen species that are able to kill cancer cells. The results showed that both formulations induce significant anticancer effects, with IC_50_ values of 65.80 µg/mL for the pure nanoparticles and 44.83 µg/mL for the ionic liquid nanoparticles [221]. Additionally, the praseodymium-doped zinc oxide nanoparticles exhibit a dose-dependent cytotoxic effect against human breast cancer cells (MDA-MB-231), with IC_50_ values varying from 7.9 µg/mL for nanoparticles containing 1% praseodymium to 9.9 µg/mL for nanoparticles containing 3% praseodymium [232]. Due to its interconfigurational transition from [Xe] 4f^1^5d^1^ to [Xe] 4f^2^, praseodymium +3 can convert X-rays into UVC radiation. This property was used by Müller et al., who synthesized UVC-emitting LuPO_4_:Pr^3+^ nanoparticles and tested their effects in A549 lung cancer cells, as UV radiation directly targets cell DNA. Although the compound did not show cytotoxic activity, it delayed the growth of the cancerous cells. When combined with X-rays, the nanoparticles reduced radiation resistance, thus increasing the cytotoxic effect [233]. Finally, praseodymium can be used in combination with other lanthanide-based nanoparticles, as was the case with lutetium phosphate nanoparticles (LuPO_4_:Pr^3+^,Nd^3+^) that were doped with praseodymium (1%) and neodymium (1.5%). The role of Praseodymium was to enhance the anti-cancer activity of UVC-emitting lutetium phosphate. The results show that the formulation, in combination with ionizing radiation, significantly inhibits the growth of A495 lung cancer cells [234].

Praseodymium itself, or more precisely, its ^142^Pr radioisotope, has the ability to emit *β* and γ radiation. It can be utilized in brachytherapy, as it possesses high beta end-point energy but penetrates the tissue limitedly, causing minimal damage to healthy tissue. It has been used in the treatment of superficial cancers, such as uveal melanoma [235]. Furthermore, based on Pr^3+^ radiation properties, praseodymium could be used in X-ray-mediated photodynamic phototherapy. Hence, Mendl synthesized radioluminescent praseodymium nanoparticles with the chemical formula NaLuF4:Pr^3+^. As the complex’s emission spectrum overlaps with the absorption spectrum of the fluorescent protoporphyrin IX photosensitizer, this praseodymium-doped nanoformulation could be used for the activation of protoporphyrin. In this context, U251 human glioblastoma cells were treated with increasing concentrations (50, 100, and 200 µg/mL) of the compound, with and without the presence of protoporphyrin IX accumulation inducer (5-aminolevulinic acid). In all experiments, the formulations were exposed to different concentrations of X-ray radiation. The results showed that nanoparticles significantly increase ROS levels and, although the cytotoxic effects were variable after the 24 h treatment, a stronger reduction of viability was observed after 48 h of stimulation. After performing the clonogenic assay, a drastic reduction in viability was observed. Also, it should be noted that treatment with nanoparticles at a 200 µg/mL concentration, together with 5-ALA and 2 Gy radiation, caused the same effects at the cellular level as 20 Gy radiation alone. Moreover, the nanoparticles reduced the level of mutant p53, Nrf2, NFκB, Grp94, PARP1, and lamin B [236].

## 18. Promethium

Promethium (Pm), with Z = 61, exists in both +2 and +3 oxidation states (Table 2). Although it has 38 isotopes, none of them are stable, being the only lanthanide with this property [237], and the least studied one because of its radioactive nature [238].

Also, some authors suggest that the application of promethium is limited due to its high radioactivity [119], while others mention that it can be used as a theranostic radionuclide agent because of its favorable *β*^−^decay characteristics and γ-emission [239]. For example, Mohsin et al. tested conventional and pretargeted radioimmunotherapy in mice bearing LS174T colon tumors, as promethium emits radiation that can be absorbed into malignant tissues (Figure 4). A CC49 monoclonal antibody labeled with promethium and MeO-DOTA (conventional) and promethium and DOTA-labeled biotin, pretargeted with CC49-streptavidin, were used. Both approaches prolonged the time of tumor progression and showed no healthy tissue toxicity, thus demonstrating promising antitumor efficacy [240]. Another study has described the very high binding of ^149^Promethium-labeled trastuzumab-polyethylenimine in SKBr3 breast adenocarcinoma, but without further testing the cytotoxic effects. The binding was of a non-specific nature, explained by the internalization of the compound into the cancer cells. Nevertheless, the Kd constant was higher in the case of Promethium-labeled compounds compared to Trastuzumab alone [241]. Alternatively, Heidari et al. focused on a different method for cancer treatment. The researchers examined the characteristics of promethium nanoparticles in terms of their ability to generate heat after light stimulation, thus destroying cancerous cells. Although no in vitro or in vivo tests were conducted, it was noticed that when nanospheres and nanorods were considered, the latter were revealed to be suitable for cancer treatments due to their ability to generate more heat [242]. However, some studies suggest that the use of promethium nanoparticles is not possible, given that the oxide nanoparticles caused alveolar proteinosis when administered to laboratory animals [119], leading to an increased concentration of pulmonary surfactant, which can further cause an impaired oxygen exchange [243].

Moreover, in beagles, a detectable concentration of promethium was observed in the lungs, bones, and liver, even 5 months after the dogs were treated with promethium (III) oxide. Other studies on rats and pigs have also emphasized that promethium can accumulate in the bones. Nevertheless, it was observed in the rabbit aorta smooth muscle that promethium does not penetrate the cells, but rather desorbs from surface fibers. However, the information on its toxicokinetic properties in humans is still limited, and most of the existing studies were conducted only after environmental exposures. The available data suggest once more that promethium could be retained in the bones, but when ingested, it is not absorbed, and its radiation can be detected in the feces [237]. 

## 19. Samarium

Samarium (Sm) (Z = 62) has two oxidation states, +2 and +3 (Table 2), and is characterized by the [Xe] 4f^6^6s^2^ electron configuration [244].

Besides its coordination characteristics, samarium has caught the attention of researchers due to its ability to bind to proteins and DNA. For instance, Asadpour et al. studied the interactions between the samarium-bipyridine complex [Sm(bpy)_2_Cl_3_(OH_2_)] and Bovine Serum Albumin and salmon DNA, while also examining its anticancer activity, free or included in starch and lipid nano-encapsulated formulations, respectively. The results showed that the samarium complex binds to both albumin and DNA; the interaction with DNA occurs between the compound and the minor groove of the macromolecule. This was affirmed by molecular docking studies (lowest binding energy−7.51 kcal mol−1), KI quenching experiment (comparable quenching with and without DNA), as well as UV–vis spectroscopy (hypochromic result without shift). All three tested products, both the free complex and its two nanoformulations, exerted significant anticancer activity against breast cancer (MCF-7) and lung cancer (A549) cells, with the nanoformulations presenting higher cellular diffusion, and subsequently, a more potent antitumor effect [245]. Upon the synthesis of Samarium (III) Schiff base complexes, [Sm((E)-4-bromo-2-(((4-(2-hydroxyethyl)phenyl)imino)methyl)phenol)_2_Cl(H_2_O)]•3H_2_O and [Sm((E)-2-(((4-(2-hydroxye thyl)phenyl)imino)methyl)-4-methoxyphenol)_2_Cl(H_2_O)]•2H_2_O, Abdel Rahman et al. tested the formulation’s anticancer activity and DNA binding. Specifically, both complexes significantly reduced the viability of MCF-7 and HepG2 cancerous cells, with IC_50_ values spanning from 66 to 14 (µg/mL). Notably, the Samarium complexes showed better anticancer activity compared to Schiff bases alone. Additionally, the complexes interacted with the DNA molecule, with the binding constants of 0.72 × 10^4^ and 0.47 × 10^4^. Every complex exhibited a greater binding affinity than its free Schiff base [246]. Bao et al. also observed that the complex between samarium, alkaloid liriodenine, and picric acid, [Sm^III^(LA)_2_(pic)_3_], moderately binds to DNA in an intercalated manner, as revealed by a moderate value of the intrinsic binding constant (5.03  ×  10^3^ L·mol^–1^), weakened fluorescence intensity of the complex, and increased viscosity of the DNA solution. Additionally, the compound significantly inhibited the growth of ovarian (SK-OV-3), hepatic (HepG2), and bladder (T-24) cancer cells. With IC_50_ values of 10.01  ±  0.55, 10.76  ±  0.19, and 8.85  ±  1.12 µM, respectively, its activity was comparable to that of cisplatin while also showing the advantage of having higher solubility than both liriodenine and cisplatin [247]. The interaction between Samarium and the DNA molecule also encouraged Aziz et al. to study the effects of samarium complex [Sm(L)(NO_3_)(H_2_O)] against MCF-7 and HCT-116 cells. The IC_50_ value was 3.062 ± 1.61 µg/mL in breast cancer and 1.768 ± 0.89 µg/mL in colon carcinoma cells, thus indicating moderate toxicity. The complex showed higher potency compared to 5-fluorouracil and cisplatin, but was less effective than another tested lanthanide complex, [La(L)(NO_3_) (H_2_O)] [248]. Furthermore, the samarium-phenanthroline complex, [Sm(phen)_3_]Cl_3_, showed high cytotoxic activity when tested against T-cell acute lymphoblastic leukemia (MOLT-4), with an IC_50_ value of 0.275 mM. Using fluorescence spectroscopy and circular dichroism, the researchers also confirmed that the complex interacts with the DNA molecule through groove binding, while molecular docking studies showed the formation of hydrophobic forces between the complex and the macromolecule [249]. Another study conducted on samarium-epicatechin nanoparticles has shown that the combination does not cause toxic effects in healthy cells while significantly reducing the viability of C26 colon cancer cells. Furthermore, the complex has been shown to have fewer side effects compared to 5-fluorouracil. Also, the nanoparticles exhibited stronger anticancer activity compared to samarium or epicatechin alone, which indicates a synergistic activity between the two drugs. As an underlying mechanism, the authors proposed that the complex increases ROS production, decreases ATP concentrations, leads to a loss of mitochondrial membrane potential, and finally causes apoptosis (Figure 2) [250]. Similarly, in order to improve epigallocatechin-3-gallate’s anticancer activity against melanoma cells, samarium was used [251]. Not only did samarium exert therapeutic effects, but it also stabilized the epigallocatechin-3-gallate through cohesion. Namely, the Sm^III^-EGCG nanocomplex showed better cytotoxic effects against the B16F1-0 cell line than its individual components. While the cancerous cells died as a consequence of mitochondrial dysfunction, the healthy cells (NIH3T3, HLEC) were negligibly affected. Additionally, the formulation significantly decreased the tumor size in the mouse xenograft model, while reducing the metastasis [252]. Likewise, Samarium was used to enhance the efficiency of titanium dioxide nanoparticles as radiosensitizers in radiation therapy. The nanoparticles—(Ti(Sm)O_2_)—were tested against DU145 and A549 cell lines using four different concentrations (25, 50, 100, and 200 µg/mL). While none of the tested doses reduced the viability of the cancerous cells, a significant increase in ROS was observed after the cells were irradiated with X-rays. Also, ROS generation was higher in the case of Samarium-doped nanoparticles, compared to simple titanium dioxide formulations. Moreover, a dose-enhancement effect of the radiation was observed. Overall, the addition of Samarium improved the radiosensitizing activity of titanium dioxide [253]. Another paper described the synthesis and anticancer potential of Flumequine ligand Samarium complex [Sm(III)(FLQ)_2_(Cl)(H_2_O)]•nH_2_O. Namely, when tested against MCF-7 and HepG-2 cell lines, the compound showed promising effects [254]. Furthermore, Nabeel et al. have tested the activity of samarium-doped zinc oxide nanoparticles against the Ehrlich solid tumor, with samarium acting as an efficacy enhancer. The addition of samarium improved the anticancer activity of zinc oxide in a dose-dependent manner by elevating the affinity of the complex for the CXCR4 receptor. Moreover, due to its similarity to Ca^2+^ ionic radii, Samarium ions show a high affinity towards Ca^2+^ binding sites, thus modifying cell membrane permeability. In addition, samarium possibly prevented zinc oxide-caused hepatotoxicity while also reducing cancer cell resistance [251]. It was also noticed that samarium attributes anticancer activity to magnesium implants that can be administered to patients suffering from bone cancer. More precisely, samarium oxide was used for the coating of magnesium alloy implants, and the coating extract was tested against MG63 osteosarcoma cells. The 100% extract showed a 96% anticancer activity after 72 h of treatment, the respective effect being associated with Sm^3+^ ions [255]. Another study reported that samarium-doped hydroxyapatite nanoparticles are biocompatible with the bone-derived MC3T3-E1 cell line. Although the results were dependent on both the exposure length and dose, samarium successfully increased the cytocompatibility of hydroxyapatite [256]. A phase 2 clinical trial was conducted in order to observe the effects of Samarium-153-ethylene diamine tetramethylene phosphonate (Sm-153-EDTMP) in metastatic castration-resistant prostate cancer. It has already been shown in preclinical studies that this Samarium formulation mediates immune mediated killing, while also causing cancerous cell destruction due to beta particle emission. The study focused on 44 patients who were administered simple Sm-153-EDTMP or its combination with the PSA-TRICOM vaccine. Although the Sm-153-EDTMP treatment alone did not result in PSA reduction, the combination with the vaccine showed significant clinical improvements. The authors also mention some limitations of Sm-153-EDTMP use, like hematologic toxicity and limited effects (palliative) in clinical studies [257].

Further, Table 3 summarizes all of the effects exhibited by lanthanides, observed in vitro, in vivo, and in clinical trials. Along with specific protein and DNA binding, REE elements have shown cytotoxic activity against various cancers via ROS generation, DNA damage, apoptosis activation, mitochondrial impairment, and organelle disruption.

## 20. Molecular Mechanisms Supporting Lanthanides Anticancer Activities

**Figure 6:**
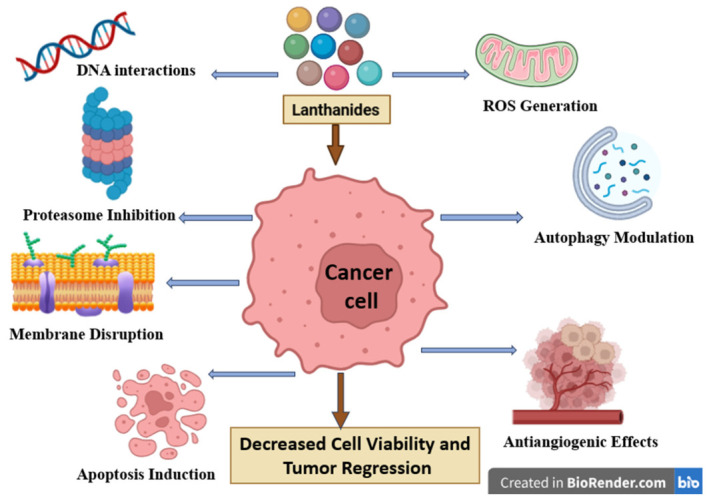
Anticancer activity of lanthanides. (Created in BioRender, 1 April 2026).

Figure 6 describes the many layered anticancer mechanisms exerted by lanthanides in breast cancer cells. One can notice that lanthanides induce their therapeutic effects through coordinated intracellular and extracellular pathways, which include the direct interaction with DNA as well as the modulation of redox homeostasis via the alteration of the GSH/GSH ratio, finally leading to oxidative stress. Lanthanides also promote the generation of reactive oxygen species, the disruption of mitochondrial functions and proteasome activity, collectively impairing essential cellular processes. Additional mechanisms of action involve cell membrane disruption, alteration of the autophagy process, and antiangiogenic effects that limit tumor vascularization. All these molecular processes result in apoptosis induction, decreased cell viability, and ultimately tumor regression. Collectively, such processes emphasize the pleiotropic nature of lanthanide-mediated cytotoxicity and indicate their potential as anticancer agents.

Although there are several anticancer molecular mechanisms shared by all rare-earth elements, each element preferentially exerts certain biological effects. Table 4 intends to further clarify the molecular mechanisms underlying the rare-earth element-mediated anticancer activity and to attribute these effects to specific lanthanides and their representative compounds. It summarizes the main mechanistic classes and also highlights their subsequent molecular events and corresponding products. An essential effect is the alteration of the redox equilibrium and oxidative stress due to excessive ROS production, lipid peroxidation (elevated MDA), and disruption of antioxidant defenses (e.g., GSH and CAT), as reported for Tb_2_O_3_, Y_2_O_3_, Yb_2_O_3_, Er_2_O_3_, Dy complexes, Eu_2_O_3_, and CeO_2_ within an acidic environment. Several REEs, such as Y_2_O_3_, Er_2_O_3_, and Dy-doped ferrites, also induce mitochondrial dysfunction and intrinsic apoptosis through the loss of mitochondrial membrane potential (ΔΨm), p53 activation, Bcl-2 downregulation, caspase-9 activation, and modulation of several respiratory chain components, such as ND3. Direct genomic effects were reported via DNA groove binding, cleavage, and ROS-mediated damage (e.g., Tb, Y, Yb, Dy complexes), while Er^3+^ uniquely promotes ROS-independent DNA condensation through phosphate backbone neutralization and helix collapse. Additional mechanisms include ferroptosis-related signaling (HO-1 upregulation by Y_2_O_3_), photodynamic cytotoxicity caused by light or X-ray-induced singlet oxygen generation (Tb, Tm_2_O_3_, Yb-porphyrins, Er(acac)TPP), and the radiosensitization that enhances DNA damage induced by radiation (Tm_2_O_3_, CeO_2_, Eu-doped systems). Furthermore, certain REEs, such as Tb- and Eu-containing compounds, modulate the adhesion and metastasis processes via cadherin disruption and MMP-2 downregulation, while CeO_2_ exhibits dual redox behavior, acting as an antioxidant at neutral pH and as a pro-oxidant within the acidic tumor microenvironment.

## 21. Conclusions

Rare-earth elements collectively exhibit significant mechanistic heterogeneity as anticancer agents, presumably due to the progressive contraction of ionic radii across their series, which results in differences in terms of coordination chemistry, redox behavior, and radiophysical properties. From lanthanum to lutetium, these elements have been investigated in diverse cancer environments where, either as coordinative complexes or metal ions, they interfered with the modulation of several intracellular signaling pathways, redox-mediated apoptosis, and DNA binding and cleavage. Early lanthanides, such as lanthanum and cerium, have been mainly investigated for their redox and oxidative stress-modulating properties, including the generation or scavenging of reactive oxygen species, depending on their respective oxidation state and nanostructure formulation. Praseodymium, neodymium, samarium, and europium have been studied in terms of interaction with DNA, photophysical parameters, and radiosensitizing potential, usually formulated as nanoparticles or coordination complexes. Gadolinium has achieved widespread clinical use as an MRI contrast agent and is currently under investigation as a theranostic and radiosensitizing agent, while terbium and holmium exhibit suitable optical and radiophysical attributes for multimodal imaging and targeted radiotherapy. Ytterbium and erbium have been exploited in photodynamic, photothermal, and upconversion-based platforms that are able to exert spatially controlled cytotoxic effects and image-guided treatment approaches. Dysprosium and thulium are primarily in the experimental phase, yet they exhibit promising ROS-mediated and DNA-targeting anticancer effects in preclinical assays. Lutetium is the most commonly used rare-earth element in clinical settings, especially in radioligand therapies that use β-emitting isotopes to target tumors.

Despite clear advances, the degree of their clinical translation varies significantly between lanthanides. Radioisotope-based nanostructures, particularly containing lutetium, have achieved widely acknowledged therapeutic application, while most non-radioactive lanthanide complexes and nanomaterials are still in experimental or early translational stages. For such emerging systems, moving towards clinical relevance will require in vivo validation in terms of pharmacokinetics and biodistribution, assessment of their metabolic fate and clearance mechanisms, as well as long-term safety evaluations, in order to avoid tissue accumulation and subsequent organ toxicity. Future research should address the optimization of the structure–activity relationship, the modulation of ligand chemistry in order to increase selectivity and stability, and their controlled integration in therapy by adhering to the current multimodal treatment approach. In addition, future developments in surface functionalization, such as the conjugation with specific ligands for targeting purposes (e.g., peptides, antibodies, aptamers), may significantly enhance tumor specificity and reduce side effects. Another future development may consist of designing systems that are able to react more promptly to specific parameters of the tumor microbiome, such as the pH, the enzymatic activity, or redox differences between normal and tumor cells. The design of stimuli-responsive or smart nanoplatforms capable of drug release in a controlled manner or activation under tumor-specific conditions stands as a promising option to improve the therapeutic outcome. Therefore, it is also interesting to use a hybrid material or extreme strata of substances whose behavior is similar to that of a small zone of reaction at the level of a cell. For example, they can be integrated with biologically inspired structures, which will result in more precise interactions. Such bioinspired systems may include membrane-coated nanoparticles, protein-based carriers, or extracellular vesicle–mimetic platforms that provide improved biocompatibility, the ability to evade the immune system, and optimized cellular uptake. Furthermore, the integration of rare-earth-based nanosystems with emerging technologies such as artificial intelligence-guided drug design and high-throughput screening could accelerate the identification of optimal therapeutic combinations. Simultaneously, efforts should also focus on the development of scalable and reproducible synthetic methods that are able to provide batch-to-batch consistency, which is essential for regulatory approval and clinical translation. All things considered, this viewpoint can lead to a more flexible and coercive therapeutic system with a greater capacity for compliance with clinical needs. Of note, the interdisciplinary collaboration between various specialists in complementary areas, such as chemists, biologists, clinicians, and materials scientists, will be essential to bridge the gap between experimental findings and clinical implementation. Such systematic and mechanistic investigations are essential in order to establish whether the diverse and chemically different lanthanide platforms can ultimately achieve clinically valuable oncological outcomes.

## Figures and Tables

**Table 1:** Comparison of key chemical and physical properties of Sc, Y, and the lanthanides.

Property	Sc	Y	Lanthanides (La–Lu)	Comparative Insight
Electronic configuration	[Ar] 3d^1^ 4s^2^	[Kr] 4d^1^ 5s^2^	[Xe] 4f^1–14^ 6s^2^ (varies by element)	Lanthanides contain 4f electrons; Sc and Y do not
Common oxidation state	+3 (dominant)	+3 (dominant)	+3 (dominant); some +2/+4	All primarily trivalent
Ionic radius	Smallest	Intermediate	Larger initially (La) → decreases across series	Lanthanide contraction
f-electrons	None	None	1–14 (partially filled 4f orbitals)	Responsible for unique lanthanide properties
Magnetic properties	Weak	Weak	Strong paramagnetism	Due to unpaired 4f electrons
Optical properties	Limited	Limited	Strong luminescence (sharp f–f transitions)	Characteristic narrow emission bands
Coordination number	6–9	6–9	6–9 (often higher)	All favor high coordination
Bonding character	Mostly ionic	Mostly ionic	Mostly ionic	Hard Lewis acids; prefer oxygen-donor ligands
Redox behavior	Very limited	Very limited	Some variability (Ce^4+^, Eu^2+^, Yb^2+^)	Lanthanides show greater redox flexibility
Geochemical occurrence	Rare	Low abundance	Occur together in REE minerals (monazite, bastnäsite)	Similar ionic radii enable substitution in mineral lattices
Abundance	Very low	Low	Moderate (La, Ce, Nd among most abundant REEs)	Lanthanides generally more abundant than Sc and Y

→ leads to/generates.

**Table 2:** Overview of the atomic and physicochemical properties of the REEs discussed in the order presented in the manuscript. Note: Data compiled from PubChem National Center for Biotechnology Information (NCBI). Periodic Table of Elements. Available online: [26] (accessed on 2 April 2026).

Element	Symbol	Electron Configuration	Atomic Number	Atomic Mass (u)	Density (g/cm^3^)	Melting Point (°C)	Boiling Point (°C)	Oxidation States	Appearance
Terbium	Tb	[Xe]6s^2^4f^9^	65	158.92	8.23	1356	3123	+3 also reported: +1, +2, +4	Silvery-gray metal
Thulium	Tm	[Xe]6^s2^4f^13^	69	168.93	9.32	1545	1950	+3 also reported: +2	Silvery-gray metal
Yttrium	Y	[Kr]5s^2^4d^1^	39	88.91	4.47	1526	2930	+3 also reported: +1, +2	Silvery lustrous metal
Scandium	Sc	[Ar]4s^2^3d^1^	21	44.96	2.99	1541	2836	+3 also reported: +1, +2	Silvery-white metal
Ytterbium	Yb	[Xe]6s^2^4f^14^	70	173.05	6.90	819	1196	+2, +3	Bright silvery metal
Cerium	Ce	[Xe]6s^2^4f^15^d^1^	58	140.12	6.77	795	3443	+3, +4 also reported: +1, +2	Iron-gray lustrous metal
Erbium	Er	[Xe]6s^2^4f^12^	68	167.26	9.07	1529	2868	+3 also reported: +1, +2	Bright, silvery, lustrous metal
Dysprosium	Dy	[Xe]6s^2^4f^10^	66	162.50	8.55	1407	2562	+3 also reported: +1, +2	Bright, silvery, lustrous metal
Europium	Eu	[Xe]6s^2^4f^7^	63	151.96	5.24	826	1529	+2, +3 also reported: +1	Silvery-white metal
Gadolinium	Gd	[Xe]6s^2^4f^7^5d^1^	64	157.25	7.90	1312	3000	+3 also reported: +1, +2	Silvery-white lustrous metal
Holmium	Ho	[Xe]6s^2^4f^11^	67	164.93	8.80	1461	2600	+3 also reported: +1, +2	Bright, silvery, lustrous metal
Lanthanum	La	[Xe]6s^2^5d^1^	57	138.91	6.15	920	3464	+3 also reported: +1, +2	Silvery-white metal
Lutetium	Lu	[Xe]6s^2^4f^14^5d^1^	71	174.97	9.84	1652	3402	+3 also reported: +1, +2	Silvery-white metal
Neodymium	Nd	[Xe]6s^2^4f^4^	60	144.24	7.01	1024	3074	+3 also reported: +2, +4	Bright, silvery, lustrous metal
Praseodymium	Pr	[Xe]6s^2^4f^3^	59	140.91	6.77	935	3130	+3 also reported: +5, +4, +2	Silvery metal
Promethium	Pm	[Xe]6s^2^4f^5^	61	144.91	7.26	1042	3000	+3 also reported: +2	Radioactive metallic element
Samarium	Sm	[Xe]6s^2^4f^6^	62	150.4	7.52	1072	1900	+2, +3 also reported: +1, +4	Bright, silvery, lustrous metal

**Table 3:** General overview of REE metals’ biological effects, with a specific focus on mechanisms underlying their anticancer activity in different cancerous cell lines.

REE	Cell Line		Anti-Cancer Effects/Other Effects	Reference
**Terbium**	Breast cancer	MCF-7	Selective cytotoxicity	[23,32]
		4T1-luc	↑ ROS levels, DNA damage → tumor cell death	
	Glioblastoma	U-251 MG	radical species generation	[27]
	Osteosarcoma	MG-63 and Saos-2	↑ ROS generation → oxidative stress, DNA damage, nuclear fragmentation, and the activation of apoptotic pathways	[34]
	Colon cancer	HT29	Selective cytotoxicity	[29]
	Lung cancer	A-549	Selective cytotoxicity	[23]
	Melanoma	B16-F10 murine	Selective cytotoxicity	[29]
	Hepatic cancer	Hep-G2	Selective cytotoxicity	[29]
	Healthy cells	Human fibroblasts	No cytotoxicity	[23]
	Binding to DNA and bovine serum albumin; strong antioxidant activity toward hydroxyl radicals (•OH) and superoxide anions (O_2_^−•^); ROS generation Interaction with the Ca^2+^-binding pockets of classical cadherins by mimicking calcium ions; binding to specific sites in proteins such as calmodulin, ion channels, and Ca^2+^-ATPases; induction of transcriptomic and signaling alterations, including the regulation of genes involved in processes like apoptosis, inflammation, and responses to cellular stress; decreasing the levels of dopamine and serotonin			[23,28,30,31,33,35,36,37,38,45,46,47]
**Thulium**	Breast cancer	4T1	ROS generation	[55,56]
		Breast tumor in mice	ROS generation	[55]
	Metastatic skin squamous cell carcinoma	Patient-derived cells	Radio sensitizer	[54]
	Gliosarcoma	9LGS	Radio sensitizer	[58]
	Healthy cells	L929	Low cytotoxicity	[56]
**Yttrium**	Breast cancer	MDA-MB-231	↑ ROS, ↑ malondialdehyde (MDA), ↓ glutathione (GSH) activity, ↓ catalase activity (CAT), induction of oxidative stress accompanied by antioxidant response; improved both early and late apoptosis; ↑ CASP3 and CASP8 (pro-apoptotic markers), ↓ anti-apoptotic gene Bcl2	[77]
		MCF-7	Cytotoxic effects	[74]
	Renal cancer	Caki-2	Apoptosis	[78]
	Hepatic cancer	HepG2	↑ ROS, loss of mitochondrial membrane potential, ↑ p53 and mitochondrial ND3, ↓ Bcl-2 → intrinsic apoptotic pathway	[80]
	Lung cancer	A549	Cytotoxic effects	[74]
	Skin cancer	A-431	Apoptosis, ↑ ROS, loss of mitochondrial membrane potential, overexpression of p53, and ND3; Bcl-2 reduction	[79]
	Pancreatic cancer	PANC	Increased levels of apoptotic and necrotic cells, ROS generation, and DNA fragmentation; the expressions of p53, ND3, and Bcl-2 were regulated in a coordinated manner; ↑ ROS, loss of mitochondrial membrane potential, overexpression of p53, and ND3; Bcl-2 reduction	[81]
	Healthy cells	HDF	↑ GSH and MDA, CAT unchanged	[77]
		HDF	Minimal changes in viability	[79]
		Kidney epithelial cells (MDCK)	No substantial cytotoxicity	[78]
		Retinal pigment epithelial cells (REP1	Minimal toxicity	[77]
	Strong bonding with DNA and bovine serum albumin			[74]
**Scandium**	Breast cancer	MDA-MB-231	Cytotoxic effects	[93,94]
	Prostate cancer	DU145	Cytotoxic effects	[93]
	Osteosarcoma	MNNG/HOS	Cytotoxic effects	[94]
	Melanoma	A375	Cytotoxic effects	[94]
	Lung cancer	A549	Cytotoxic effects	[94]
	Glioblastoma	U251	Cytotoxic effects	[94]
	Colon cancer	Caco-2	Cytotoxic effects	[94]
		HCT116	Relatively non-toxic	[95]
		HT-29	Relatively non-toxic	[95]
**Ytterbium**	Cervical cancer	HeLa cells	ROS formation	[109]
	Breast cancer	MCF-7	ROS generation, metal cations release, formation of free radicals → disruption of cell membranes, mitochondria destruction, and cellular death DNA damage, oxidative stress, and disruption of cellular functions	[110,111]
	Lung cancer	A-549	DNA damage, oxidative stress, and disruption of cellular functions	[111]
	Significant binding to fish DNA and BSA			[111]
**Cerium**	Breast cancer	MCF-7	Apoptosis and inhibited cell proliferation	[119,124]
		AMJ13	Selective cytotoxicity	[122]
	Cervical cancer	HeLa	Apoptosis and inhibited cell proliferation	[119]
	Ovarian cancer	Multiple cell lines	↓ basal ROS levels; inhibition of growth factor-induced migration and invasion (SDF1, HB-EGF, VEGF165, and HGF)	[127]
		Nude mice bearing ovarian xenografts	Decreased Ki-67 expression and reduced tumor angiogenesis	[127]
	Lung cancer	A549	Apoptosis and inhibited cell proliferation	[119]
		H460	Pro-oxidant effect; significant increase in ROS; apoptosis	[121]
	Prostate cancer	LNCaP	Apoptosis	[124]
	Liver cancer	HepG2	Apoptosis and inhibited cell proliferation	[119]
	Colon cancer	HT-29	Apoptosis by BAX and Caspase-1 gene activation, blocking of the tumor protection gene Bcl-2	[123]
	Pancreatic cancer	L3.6pl and Panc1	Cytotoxic effects	[126]
		In vivo using athymic mice	Increases in the activation of both JNK and caspase 3	[126]
	Brain cancer	AMGM5	Selective cytotoxicity	[122]
	Esophageal cancer	YM1	↓ ROS, ↓ malondialdehyde (MDA); ↑ superoxide dismutase (SOD), catalase (CAT), thiols, and total antioxidant capacity (TAC)	[120,122]
	Cancer stem cells	CSC-LC	↓ ROS, ↓ malondialdehyde (MDA); ↑ superoxide dismutase (SOD), catalase (CAT), thiols, and total antioxidant capacity (TAC)	[120]
	ORL cancers	A253 SCC-25 FaDu	Cytotoxic effects	[125]
	Healthy cells	REF	No effects	[122]
**Erbium**	Lymphoma cancer	U937	↑ ROS generation; DNA and mitochondrial membrane potential damage; dysregulation of apoptotic (*p53*), anti-apoptotic (*Bcl2*) and mitochondrial *ND3* gene expression → apoptosis and necrosis	[132]
	Liver cancer	HepG-2	↑ ROS, DNA fragmentation, apoptotic effect, cytotoxic effects	[133,138]
	Breast cancer	MCF-7 cell line	Cytotoxic effects	[134,137,138]
		MDA-MB-231	Cell death possibly caused by apoptosis via internalization	[135]
	Lung cancer	H1299	Selective cytotoxicity	[136]
		A549	Selective cytotoxicity	[136]
		H460	Selective cytotoxicity	[136]
	Squamous cancer	NCI-H226	Selective cytotoxicity	[136]
	Cervical cancer	HeLa	Selective cytotoxicity	[136,137]
	Colon cancer	HCT116	Selective cytotoxicity	[136]
	Healthy cells	Beas2b	No effects	[136]
		Vero cells	No effects	[137]
	Renal carcinoma	786-O	Selective cytotoxicity	[136]
	Interactions with double-stranded DNA			[139]
**Dysprosium**	Melanoma	A375	Nuclear condensation and fragmentation, ↑ Caspase-9 → activation of the intrinsic apoptotic pathway	[143]
	Breast cancer	MCF-7	Nuclear condensation and fragmentation, ↑ Caspase-9 → activation of the intrinsic apoptotic pathway; selective cytotoxicity	[143,149]
	Cervical cancer	HeLA	Selective cytotoxicity	[149]
	Colorectal cancer	HCT-116	Cytotoxicity at higher doses	[144]
			Production of oxygen species, interactions with the DNA	[148]
	Lung adenocarcinoma	A549	Cytotoxic effects	[146]
	Prostate carcinoma		Cytotoxic effects	[147]
	Promyelocytic leukemia		Cytotoxic effects	[147]
	Liver cancer	Hep-G2	Selective cytotoxicity	[149]
		BEL-7404	Selective cytotoxicity	[149]
	Healthy cells	HL-7702	No effects	[149]
**Europium**	Breast cancer	MCF7 and	Cytotoxic effects	[157]
		4T1	Cytotoxic effects	[157]
	Melanoma	A375	Selective cytotoxicity	[158]
		Murine melanoma model	Selective cytotoxicity	[158]
	Cervical cancer	HeLa	Selective cytotoxicity	[158,258]
	Healthy cells	L02	No effects	[158]
	Osteosarcoma cells	143B	Cytotoxic effects	[154]
		K7M2	↓ matrix metalloproteinase-2 (MMP-2)	[155]
	Lung adenocarcinoma	A549	Cytotoxic effects	[258]
	Lung cancer	A549	Most likely attributed to ROS generation	[156]
**Gadolinium**	Cervical cancer	HeLa	Block of the final phases of autophagy, phagosome accumulation	[162]
	Lung cancer	A549	↑ ROS levels, apoptosis by affecting the Bax and Bcl-2 protein expression, inhibited tumor cell migration, arrest in the G0/G1 phase	[165]
	Nasal squamous cell carcinoma	RPMI 2650	Cytotoxic effects	[163]
		CNE-1	Cytotoxic effects	[163]
	Prostate cancer	22Rv1 tumor-bearing mice	↑ Bax, p53, and γ-H2AX. ↓ PCNA and Bcl-2 → promotion of apoptosis and preventing tumor growth; cellular death and fragmentation; DNA damage	[166]
	Glioblastoma multiforme		Anti-cancer activity	[167]
	Melanoma	Mice bearing subcutaneous B16F10 melanoma	Cytotoxic effects as a neutron capture therapy agent	[168]
	Apoptosis, ferroptosis, and ferroptosis-induced immune response			[164]
**Holmium**	Liver cancer	Liver tumour	Radiation effects	[171]
		Hepa1–6	Less pronounced cytotoxic effects	[174]
	Lung cancer	A549	Minimal cytotoxicity when administered alone	[172]
	Breast cancer	MCF-7	Oxidative stress induced by ROS, calcium transport disruption, direct interactions with cellular organelles and DNA, endoplasmic reticulum stress, and activation of MAPK pathways	[173]
		3T3	Oxidative stress induced by ROS, calcium transport disruption, direct interactions with cellular organelles and DNA, endoplasmic reticulum stress, and activation of MAPK pathways	[173]
		in ovo	Inhibition of new blood vessel development	[173]
		4T1	Apoptosis and necrosis	[174]
	Healthy cells	HUVEC	No cytotoxic effects	[174]
	Renal cancer	Orthotopic mouse model	Radiation → necrosis, infiltrations of inflammatory cells, and extensive tumor cell death	[175]
	Osteosarcoma	MC3T3-E1	Reduced viability of cancerous cells, not caused primarily by holmium	[176]
		MG-63	Reduced viability of cancerous cells, not caused primarily by holmium	[176]
**Lanthanum**	Liver	SMMC-7721	Proliferation stop, apoptosis by blocking the Hedgehog pathway. ↓ Gli1 and Sonic hedgehog, Cyclin D1 and Bcl-2; ↑ p21 and cleaved Caspase-3	[178]
	Ovarian cancer	SKOV3	Block of the PI3K/Akt pathway, reduction of RAD51 expression, dysregulated DNA repair; apoptosis, with higher Bax and cleaved caspase-3 and lower Bcl-2	[179]
	Glioblastoma		Near-infrared light: ↑ ROS → oxidative and photothermal tumor suppression	[181]
	Osteosarcoma	MG63	Selective cytotoxicity	[180]
	Healthy cells	Vero	Enhanced viability	[180]
**Lutetium**	Neuroendocrine tumors	Neuroendocrine tumors trial	Peptide receptor radionuclide therapy (PRRT) targeting somatostatin receptors	[195]
	Prostate cancer	Metastatic castration-resistant prostate cancer trial	Binds to PSMA on the surface of tumor cells → internalization → β radiation generates DNA double-strand breaks → apoptotic cell death	[198,199]
	Breast cancer	HER2-positive models	Cytotoxic activity	[203]
		CT26-FAP tumor cells, in vivo models	Increasing the sensitivity to PD-1/PD-L1 immunotherapy; enhanced CD8^+^ T-cell infiltration, increased immunogenic cell death	[202]
**Neodymium**	Liver cancer	HepG2	Apoptosis, ↑ pro-apoptotic genes, p53, Bax, and caspase-3; ↓ anti-apoptotic gene Bcl-2; ↑ ROS, changes in cell-cycle distribution.	[217]
		Liver cells	Oxidative and genotoxic stress	[217,218]
	Lung cancer	A549	Apoptosis, ↑ pro-apoptotic genes, p53, Bax, and caspase-3; ↓ anti-apoptotic gene Bcl-2; ↑ ROS, changes in cell-cycle distribution.	[217]
		Lung cancer cells	Oxidative and genotoxic stress	[217,218]
	Breast cancer	MCF-7	Condensation of the DNA helix, apoptosis; disruption of the cytoskeleton, alterations in nuclear structure, heightened ROS production, caspase-3 activation, alterations in mitochondrial membrane permeability, and DNA fragmentation	[215,218]
		Breast cancer cells	Interaction with DNA, DNA helix condensation, apoptosis	[215]
	Melanoma	A375	Condensation of the DNA helix, apoptosis; disruption of the cytoskeleton, alterations in nuclear structure, heightened ROS production, caspase-3 activation, alterations in mitochondrial membrane permeability, and DNA fragmentation	[218]
	Pancreatic cancer	PANC	Condensation of the DNA helix, apoptosis; disruption of the cytoskeleton, alterations in nuclear structure, heightened ROS production, caspase-3 activation, alterations in mitochondrial membrane permeability, and DNA fragmentation	[218]
	Healthy cells	HaCaT	No toxic effects at lower concentrations	[218]
	DNA binding, induction of oxidative stress, mitochondrial dysfunction, and photothermal or imaging-assisted therapy optimization			[215,217]
**Praseodymium**	Melanoma	A375	Depending on the tested compound, no cytotoxic effects or cytotoxic effects	[224,228]
	Liver cancer	HepG-2,	Depending on the tested compound, no cytotoxic effects or cytotoxic effects	[224,227]
	Breast cancer	MCF-7,	Depending on the tested compound, no cytotoxic effects or cytotoxic effects: A rise in ROS generation, subsequent apoptosis and DNA fragmentation, binding with DNA; targeting of lysosomal activity, mitochondrial metabolism alteration; influencing the zinc-mediated toxicity: it promotes Zn^2+^ influx into the cell, thus disrupting cellular survival processes	[137,221,224,228,259]
		MDA-MB-231	Depending on the compound, no cytotoxic effect or cytotoxic effects: targeting of lysosomal activity, mitochondrial metabolism alteration; influencing the zinc-mediated toxicity: it promotes Zn^2+^ influx into the cell, thus disrupting cellular survival processes	[224,232,259]
	Lung cancer	A549	Depending on the tested compound, no cytotoxic effects or cytotoxic activity, delay in the growth of the cancerous cell	[224,233]
	Neuroblastoma		cytotoxic effects	[226]
	glioblastoma	U251	↑ ROS levels, ↓ mutant p53, Nrf2, NFκB, Grp94, PARP1 and lamin B	[236]
	Cervical cancer	HeLa	A rise in ROS generation, subsequent apoptosis and DNA fragmentation, binding with DNA; targeting of lysosomal activity, mitochondrial metabolism alteration; influencing the zinc-mediated toxicity: it promotes Zn^2+^ influx into the cell, thus disrupting cellular survival processes	[137,259]
	Colon cancer	HCT-116	Signs of chromatic fragmentation and nuclear disintegration, suggesting apoptosis.	[229]
		HCT116 *p53 KO,* HCT116 WT, HCT116 *Bax/Bak DKO*	Targeting of lysosomal activity, mitochondrial metabolism alteration; influencing the zinc-mediated toxicity: it promotes Zn^2+^ influx into the cell, thus disrupting cellular survival processes	[259]
		HT-29	Cytotoxic effects	[228]
	Bladder cancer	T-24	No cytotoxic effects	[224]
		HEK-293	Decreased viability	[229]
	Prostate cancer	PC-3	No cytotoxic effects	[224]
**Promethium**	Colon cancer	Mice bearing LS174T colon tumors	Radiation emission	[240]
**Samarium**	Breast cancer	MCF-7	Cytotoxic effects	[245,246,248,254]
	Lung cancer	A549	Cytotoxic effects	[248]
		A459	ROS increase without cytotoxicity	[253]
	Hepatic cancer	HepG2	Cytotoxic effects	[246,254]
	Bladder cancer	T-24	Cytotoxic effects	[247]
	Ovarian cancer	SK-OV-3	Cytotoxic effects	[247]
	T-cell acute lymphoblastic	MOLT-4	Interacts with the DNA molecule	[249]
	Colon cancer	C26	↑ ROS production, ↓ ATP concentrations, loss of mitochondrial membrane potential, apoptosis	[250]
		HCT-116	Cytotoxic effects	[248]
	Melanoma	B16F10 cell line,	Mitochondrial dysfunction	[252]
		Mouse xenograph model	Mitochondrial dysfunction	[252]
	Osteosarcoma	MG63	Cytotoxic effects	[255]
	Prostate cancer	Patients with metastatic castration-resistant prostate cancer	No significant effects when administered alone	[257]
		DU145	ROS increase without cytotoxicity	[253]
	Ehrlich solid tumour		High affinity towards Ca^2+^ binding sites	[251]
	Healthy cells	Bone-derived MC3T3-E1	No Cytotoxic effects at lower doses and shorter exposure	[256]
	Binding to both albumin and DNA			[245,247]

↑—increase/upregulation; ↓—decrease/downregulation; → leads to/generates.

**Table 4:** Molecular mechanisms supporting lanthanides’ anticancer activities.

Mechanistic Class	Molecular Events	Representative REE Systems	References
Redox imbalance and oxidative stress	ROS overproduction, lipid peroxidation (↑ MDA), antioxidant dysregulation (GSH, CAT)	Tb_2_O_3_, Y_2_O_3_, Yb_2_O_3_, Er_2_O_3_, Dy complexes, Eu_2_O_3_, CeO_2_ (acidic pH)	[34,77,109,132,143,155]
Mitochondrial dysfunction and intrinsic apoptosis	ΔΨm loss, p53 activation, Bcl-2 downregulation, caspase-9 activation, ND3 upregulation	Y_2_O_3_, Er_2_O_3_, Dy-doped ferrites	[79,80,135,143]
Direct DNA targeting	Groove binding, DNA cleavage, ROS-mediated genomic damage	Tb, Y, Yb, Dy complexes	[28,74,111,144]
DNA condensation (ROS-independent mechanism)	Phosphate backbone neutralization, helix collapse	Er^3+^	[139]
Ferroptosis-related signaling	HO-1 upregulation	Y_2_O_3_	[77]
Photodynamic cytotoxicity (PDT)	Light/X-ray induced singlet oxygen generation	Tb, Tm_2_O_3_, Yb-porphyrins, Er(acac)TPP	[32,55,109,137]
Radiosensitization	Increased radiation-induced DNA damage	Tm_2_O_3_, CeO_2_, Eu-doped systems	[54,114,154]
Adhesion and metastasis modulation	Cadherin disruption (Ca^2+^ mimicry), MMP-2 downregulation	Tb, Eu systems	[31,155]
Redox-switch behavior	Antioxidant (neutral pH)/pro-oxidant (acidic TME)	CeO_2_	[118]

↑—increase.

## Data Availability

No new data were created or analyzed in this study.
